# Unraveling cell wall polysaccharides during blueberry ripening: insights into the roles of rhamnogalacturonan-I and arabinogalactan proteins in fruit firmness

**DOI:** 10.3389/fpls.2024.1422917

**Published:** 2024-09-30

**Authors:** Dayan Sanhueza, Iván Balic-Norambuena, Pablo Sepúlveda-Orellana, Sebastián Siña-López, Adrián A. Moreno, María Alejandra Moya-León, Susana Saez-Aguayo

**Affiliations:** ^1^ Centro de Biotecnología Vegetal, Facultad de Ciencias de la Vida, Universidad Andrés Bello, Santiago, Chile; ^2^ Agencia Nacional de Investigación y Desarollo (ANID) - Anillo de Investigación en Ciencia y Tecnología, Chilean Fruits Cell Wall Components as Biotechnological Resources (CHICOBIO), Santiago, Chile; ^3^ Departamento de Acuicultura y Recursos Agroalimentarios, Universidad de Los Lagos, Osorno, Chile; ^4^ Laboratorio de Fisiología Vegetal y Genética Molecular, Instituto de Ciencias Biológicas, Universidad de Talca, Talca, Chile; ^5^ ANID - Millennium Science Initiative Program, Millennium Nucleus for the Development of Super Adaptable Plants (MN-SAP), Santiago, Chile

**Keywords:** cell wall domains, polysaccharides, pectins, hemicelluloses, Rhamnogalacturonan-I, Arabinogalactan proteins

## Abstract

Blueberries (*Vaccinium corymbosum*) undergo significant texture changes during development and ripening, notably a consistent decrease in firmness, which affects fruit quality, consumer preference, transportability, and shelf life. This study examined the composition and structural modifications of the cell wall in five commercially available blueberry varieties with differing firmness levels at harvest. Our approach integrated various biochemical techniques for a comprehensive analysis of cell wall components to elucidate firmness differences at the harvest stage. One of the conclusions was the relationship between a low degree of pectin methylesterification and the presence of increased egg-box structures, which correlated with increased firmness. The data suggest that low-abundance pectins in blueberry cell walls, such as rhamnogalacturonan-I participate in firmness modulation through their side branches or by linking to arabinogalactan proteins. Additionally, the xyloglucan structure can be one of the determinants of fruit firmness. Although, this work provides a broad insight into the relationship between cell wall composition and firmness in blueberry, a more detailed analysis, specifically focusing on pectin and hemicelluloses, would be of significant value.

## 1 Introduction

Blueberries (*Vaccinium corymbosum*) undergo noticeable texture changes during development and ripening, including a consistent decrease in firmness (Giongo et al., 2013; Chen et al., 2015). Fruit firmness is a critical attribute because it directly impacts fruit quality, consumer preferences, transportability, and shelf life. Consumers highly value firmness as it closely correlates with the fruit’s freshness and overall quality (Saftner et al., 2008; Blaker and Olmstead, 2014; Gilbert et al., 2015).

The process of fruit softening naturally occurs as berries develop and ripen and continues after harvest (Cappai et al., 2018). The primary factors influencing firmness are believed to be fruit morphology, turgor pressure, enzyme activity, internal cell structure, and variations in cell wall composition (Brummell, 2006; Chea et al., 2019; Olmedo et al., 2021; Li et al., 2021). Notably, there is considerable variability in firmness levels during ripening among different blueberry varieties and even within the same crop (Beaudry et al., 1992; Lobos et al., 2014; Cappai et al., 2018). Because of its significance, the texture changes that occur in fruit ripening and postharvest have been widely studied. Textural changes are linked to the presence and structural integrity of the plant cell wall, a dynamic structure that undergoes modifications based on the fruit’s developmental stage (Nunan et al., 1998; Brummell et al., 2004; Zhang et al., 2020; Su et al., 2022). The few studies addressing blueberry softening have focused primarily on changes associated with cell wall metabolism. Unlike other fruits, hemicellulose (HC) modifications may play a more important role than pectins in texture changes during blueberry development (Angeletti et al., 2010; Vicente et al., 2007; Olmedo et al., 2021).

PCWs are highly complex, varying in polysaccharide content and structure. They are composed of a blend of polysaccharides, including cellulose, HCs, and pectins, as well as lipids, phenolic compounds, and proteins like arabinogalactan proteins (AGPs) (Showalter, 1993; Fry, 2004; Voragen et al., 2009; Scheller and Ulvskov, 2010). These polysaccharides often cross-link, forming a complex network that gives the cell wall its unique mechanical and permeability properties (Epstein, 1973; Whitney et al., 1999; Voragen et al., 2009; Höfte and Voxeur, 2017). Pectins, primarily composed of galacturonic acid (GalA), can be categorized into several domains, including homogalacturonan (HG), xylogalacturonan (XGA), rhamnogalacturonan-I (RG-I), and rhamnogalacturonan-II (RG-II) (Mohnen, 2008; Caffall and Mohnen, 2009; Zhang et al., 2021). HCs represent a group of complex carbohydrates, including β-(1-3,1-4)-glucan, xyloglucan, xylans, mannans, among others. These HCs intermingle with cellulose and pectins in the cell wall (Scheller and Ulvskov, 2010).

Early ripening changes include the degradation of the galactan/arabinan side chains of RG-I, the demethylesterification of HG, and the depolymerization of HCs. Pectin solubilization increases during ripening, but pectin depolymerization is typically most pronounced late in the ripening process. There is considerable variation in the extent of pectin depolymerization and galactan/arabinan loss among species (Brummell, 2006). During fruit ripening, several linkages connecting polysaccharides are degraded, releasing high-molecular-weight polymers (Brummell, 2006). Modification of cell wall polysaccharides can alter fruit firmness through the depolymerization of matrix glycans, changes in neutral sugar composition, and the loss of the middle lamella. During ripening, the reduction of specific sugars such as xylose, galactose, and arabinose has been observed in many crop species, including blueberries, and is associated with changes in fruit texture (Brummell, 2006; Vicente et al., 2007; Chea et al., 2019). These changes are closely linked to the combined action of cell-wall-degrading and remodeling enzymes.

To date, numerous enzymes have been studied to determine their role in regulating fruit softening. Research on tomatoes has included developing transgenic plants to silence genes encoding polygalacturonase (PG) (Sheehy et al., 1988; Smith et al., 1988; Wang et al., 2018), pectinesterase (Tieman and Handa, 1994), galactanase (TBG) (Smith et al., 2002), xyloglucan endo-transglycosylase (Maclachlan and Brady, 1994), and expansin (Brummell et al., 1999). These experiments resulted in only minor changes in the texture of the transgenic fruits. More recently, the silencing of PL in tomatoes has been shown to inhibit fruit softening (Uluisik et al., 2016; Wang et al., 2019). However, in strawberries, a non-climacteric fruit, the suppression of PG, pectate lyase (PL) (Jiménez-Bermúdez et al., 2002; Figueroa et al., 2008; Quesada et al., 2009; Posé et al., 2015), or rhamnogalacturonan lyases (Ric-Vargas et al., 2024) led to significantly firmer fruits. Thus, pectin degradation is a key process that influences the softening of fleshy fruits (Ric-Vargas et al., 2024).

Regarding studies on blueberry softening, the focus primarily revolves around changes related to cell wall metabolism. In contrast to other fruits, it appears that HC depolymerization and arabinose loss may play a more important role than pectins in affecting texture during blueberry development (Vicente et al., 2007; Angeletti et al., 2010). Traditionally, fruit firmness has been associated with the HG content, calcium presence, and their ability to cross-link and form the “egg-box” structures that create micro-gels within the cell wall, widely recognized as a primary contributor to firmness (Yamaki et al., 1983; Liu et al., 2009; Zhang et al., 2018, 2019). Notably, the application of calcium has been found to enhance both the firmness and quality of blueberries during postharvest, as reported in various studies (Angeletti et al., 2010; Beaudry et al., 2016). According to Angeletti et al. (2010), this quality improvement may result from the speculated reduction in pectin solubilization due to increased calcium content, while HC in blueberry remains unaffected. Vicente et al. (2007) reported that blueberries (cv. Duke) maintain a high GalA content that remains constant throughout ripening, and no reduction in the size of the HG pectic GalA-enriched polymers was observed. The absence of changes in GalA or in the HG polymer size does not necessarily imply that they are not involved in fruit firmness, as other factors such as the degree of methylation and the effect of RG-I and AGPs have yet to be explored.

This research investigated the composition and structural modifications of the cell wall in five blueberry varieties with different firmness levels across three developmental stages: pre-veraison, veraison, and harvest. Initially, the overall sugar composition in the total alcohol-insoluble residues (AIRs) was analyzed. Significant alterations in certain monosaccharides among the varieties were detected through the AIR analyses. To obtain a more specific compositional analysis, a chemical fractionation of AIR was conducted, resulting in the extraction of HC- and pectin-enriched fractions. The results consistently revealed variations in Ara/Rha and Gal/Rha ratios among varieties, indicating differences in the branching of RG-I polymers. To validate this, the RG-I was enzymatically separated from RG-II and HG domains, and individual sugar measurements confirmed modifications in RG-I branching. Our study aligns with previous research that identified HG and xyloglucan as key domains of cell wall polysaccharide involved in fruit firmness. However, we also observed variations in RG-I side branches and AGPs that, depending on the variety, could potentially explain the differences in blueberry firmness.

## 2 Materials and methods

### 2.1 Plant material

This study utilized five commercial highbush blueberry varieties of *V. corymbosum*, each differing in firmness: Brigitta, Draper, Duke, Legacy, and Bluecrop (**Supplementary Figure 1**). The fruit was harvested from commercial orchards located in Osorno, Chile (40°34′00′′S 73°09′00′′W; Región de Los Lagos) between November and January 2019–2020, corresponding to the standard harvest season in the region. The berries were segregated into three development stages: pre-veraison, veraison, and harvest, according to the respective producer’s commercial standards, primarily based on berry pigmentation as maturity indicator (**Supplementary Figure 1**).

#### 2.1.1 Determination of Blueberry firmness

Berry firmness was assessed for each sample, following the methodology described by Olmedo et al. (2021) with some modifications. A texture profile analysis (TPA) was performed using the TA.XT plus texture analyzer (Stable Micro Systems Ltd., Godalming, United Kingdom) equipped with an SMS P/35 flat probe (2.5 cm × 2.5 cm). This analysis utilized the lateral compression method, measuring the force on the sagittal side of the fruit. The instrumental settings included two compressions of 30% of the berry width, a test speed of 60 mm min^–1^, a post-test speed of 300 mm min^–1^, a 0.03-min interval between compressions, an auto-force trigger of 5 g, and stopping the plot at the target position. Data were acquired with a resolution of 500 pps (points per second), with 25 random berries of each variety used for analysis.

#### 2.1.2 AIR preparation

To prepare the AIR, the berries were ground in liquid nitrogen and subjected to two extractions. The first involved an overnight incubation followed by an 8-h incubation in 80% ethanol with agitation at room temperature (RT). Lipids were then removed by rinsing the extract twice for 2 h with methanol:chloroform (1:1, v/v) and twice for 1 h with acetone. The final AIR was dried overnight at RT.

#### 2.1.3 Cell wall fractionation

Different fractions of cell wall components were obtained from the AIR through sequential chemical extraction. Pectins were extracted by incubating the AIR with 0.5 M imidazole, pH 7.0, at RT, followed by two incubations with 0.2 M ammonium oxalate, pH 4.3, at 60°C. The resulting supernatants were dialyzed against water and freeze-dried. After dialysis, the pectic fractions obtained with imidazole and ammonium oxalate were combined into a single pectic fraction. The remaining pellet from pectin extraction was used to isolate HCs by rinsing it with water and then incubating it overnight at 37°C on a shaker with 6 M NaOH/1% NaBH_4_. This process was repeated twice, and the collected supernatants were dialyzed and freeze-dried.

#### 2.1.4 Acid hydrolysis of AIR, pectin, and hemicellulose and monosaccharide quantification by HPAEC-PAD

Samples were hydrolyzed for 1 h using 400 μL of 2 M trifluoroacetic acid (TFA) at 121°C. After hydrolysis, TFA was then evaporated at 45°C using nitrogen. The samples were rinsed twice with 400 μL of 100% isopropanol. The samples were resuspended in 1 mL of MilliQ water, sonicated for 15 min, centrifuged for 1 min at maximum speed, and finally filtered through a syringe with a pore size of 0.22 μm into a new tube before being analyzed using HPAEC-PAD. Inositol and allose (250 µM each) were utilized as internal controls for the TFA hydrolysis.

Monosaccharide quantification was performed using a Dionex ICS3000 ion chromatography system equipped with a pulsed amperometric detector, a CarboPac PA1 (4×250 mm) analytical column, and a CarboPac PA1 (4×50 mm) guard column as published in Sanhueza et al. (2024). For the separation of neutral sugars, the system was set at 32°C with a flow rate of 1 mL min^–1^ using a 20 mM NaOH isocratic gradient for 24 min. Subsequently, for the separation of acidic sugars, a solution of 75 mM NaOAc and 150 mM NaOH was employed for 20 min at a flow rate of 1 mL min^–1^ at 32°C. A final wash step using 200 mM NaOH for 5 min was performed after each run, followed by column equilibration with 20 mM NaOH for 6 min. Quantification relied on standard curves established for both neutral sugars [fucose (Fuc), Rha, Ara, Gal, Glc, Xyl, and Man] and acidic sugars [GalA and glucuronic acid (GlcA)].

#### 2.1.5 Quantification of the methanol content

The methanol content was analyzed using AIR and pectin preparations, following the method of Anthon and Barrett (2004). The AIR was resuspended in 50 μL of MilliQ water, and 50 μL of 1 M NaOH was used to saponify the mixture. For pectin, a concentration of 3 mg mL^−1^ was resuspended, and 50 μL of this solution was saponified with 50 μL of 0.2 M NaOH. Both types of samples were incubated for 1 h at 4°C on ice. Afterward, 50 μL of 1 M HCl was added to stop the saponification of the AIR, while 50 μL of 0.2 M HCl was added to stop the pectin saponification. To the AIR sample, 850 µL of MilliQ water was added to achieve a total volume of 1 mL, while to the pectin sample, 150 µL was added to reach a total volume of 300 µL. To 50 μL of the saponification mixture was added 100 μL of 200 mM Tris-HCl (pH 7.5), 40 μL of 3 mg mL^−1^ 3-methyl-2-benzothiazolinone hydrazone (MBTH), and 20 μL of 0.02 U μL^−1^ alcohol oxidase from *Pichia pastoris* (Sigma A-2404). The mixture was incubated for 20 min at 30°C. To reveal methanol content, 200 µL of sulfamic acid and ammonium ferric sulfate dodecahydrate (0.5% w/v each in water) was added to the previous mixture and incubated at RT for 20 min. Finally, 600 µL of MilliQ water was added, and the absorbance at 620 nm was measured. The methanol content was determined using a standard curve between 0 and 10 µg µL^−1^ of methanol. All experiments were performed with five technical replicates.

#### 2.1.6 Uronic acid quantification

The quantification of uronic acids in pectin was performed using the m-hydroxybiphenyl method, following the protocol outlined by Blumenkrantz and Asboe-Hansen (1973). This involved mixing 2 µL of a 3 mg mL^−1^ pectin solution with 18 µL of water and 100 µL of a 0.5% solution of N_2_B_4_O_7_•10H_2_O (borax) in sulfuric acid. The samples were incubated at 100°C for 5 min, and absorbance was recorded at 520 nm. Color development was initiated by adding 2 µL of 0.15% m-hydroxybiphenyl in 1 M NaOH solution, followed by measuring the absorbance at 520 nm after standing for 5 min at RT. Uronic acid content was determined using a standard curve based on 0.1 to 2 µg of GalA. All experiments were performed using six technical replicates.

#### 2.1.7 Pectin domains isolation

The AIR was saponified by incubation with 0.5 M Na_2_CO_3_ overnight at 4°C, followed by neutralization with acetic acid. The saponified AIR was then digested overnight at RT with 2.25 U mL^−1^ of endopolygalacturonase (endo-PG) from *Aspergillus aculeatus* (Megazyme) in pyridine:acetic acid:water (PyAW 1:10:200, v/v/v). The digestion was loaded into a BioGel P-30 column (2.5 × 57 cm) and eluted with PyAW 1:1:98 v/v/v at 1 mL min^−1^. To detect the fractions where the different domains eluted, 20 µL from each fraction was used to quantify total uronic acids. Fractions enriched in RG-I and OGAs were separately pooled and dried in a speed vacuum as published in Sanhueza et al. (2024).

#### 2.1.8 Immunodot blot assay

Immunodot blot assays were conducted using purified HC and RG-I fractions from the AIR of all five cultivars at three development stages as published in Sanhueza et al. (2024). Briefly, serial dilutions were prepared with an initial concentration of 2 µg µL^−1^. Subsequently, 0.7 µL of each dilution was blotted onto a 0.45-µm nitrocellulose membrane (Thermo Scientific, cat. no. 88018). The membranes were blocked with TBS-T (25 mM Tris, 0.15 M NaCl, and 0.1% w/v of Tween-20) supplemented with 3% w/v of powdered skim milk. All primary antibodies were diluted 1:50 in TBS-T with 1% w/v skim milk, and the membranes were incubated at RT. The 2F4 antibody was diluted in TCaNa buffer (20 mM Tris-HCl, pH 8.2, 0.5 mM CaCl_2_, and 150 mM NaCl_2_) per the manufacturer’s recommendation, followed by supplementation with 1% w/v of skim milk and 0.1% w/v of TBS-T. Next, the membranes were incubated with the appropriate secondary antibody conjugated to alkaline phosphatase, diluted 1:2,000 in TBS-T. Finally, the membranes were incubated with BCIP/NBT 1 Step chromogenic AP substrate developing solution (Thermo Scientific, cat. no. 34042). The dot signals were semi-quantified using ImageJ 1.53 software (Freeware, National Institutes of Health). A minimum of three technical replicates were used for the immunoblot.

#### 2.1.9 Immunofluorescence detection assay

Berries at the harvest stage were fixed in FAA solution (10% formaldehyde, 5% acetic acid, and 50% ethanol in water). The central sections of these berries underwent a dehydration process (Olmedo et al., 2021) using progressively stronger ethanol solutions, followed by xylol treatment, and ultimately embedding in Paraplast (Sigma-Aldrich, St. Louis, MO, USA). Subsequently, 3-μm-thick slices were prepared and subjected to incubation with the primary antibodies targeting various cell wall epitopes. Signal intensities were quantified and compared using ImageJ 1.53t software, with the results expressed as normalized fluorescence intensity per unit area.

#### 2.1.10 Statistical analysis

One-way analyses of variance (ANOVAs) were conducted with the Tukey test (significance level of 0.01). Comparisons between fruit stages for each cultivar were performed. For dot blot analysis, comparisons between samples were performed using Student’s *t*-test with *p* < 0.05.

## 3 Results

### 3.1 The different blueberry varieties show firmness reduction during ripening linked to the decrease of cell wall sugar content

**Figure 1:**
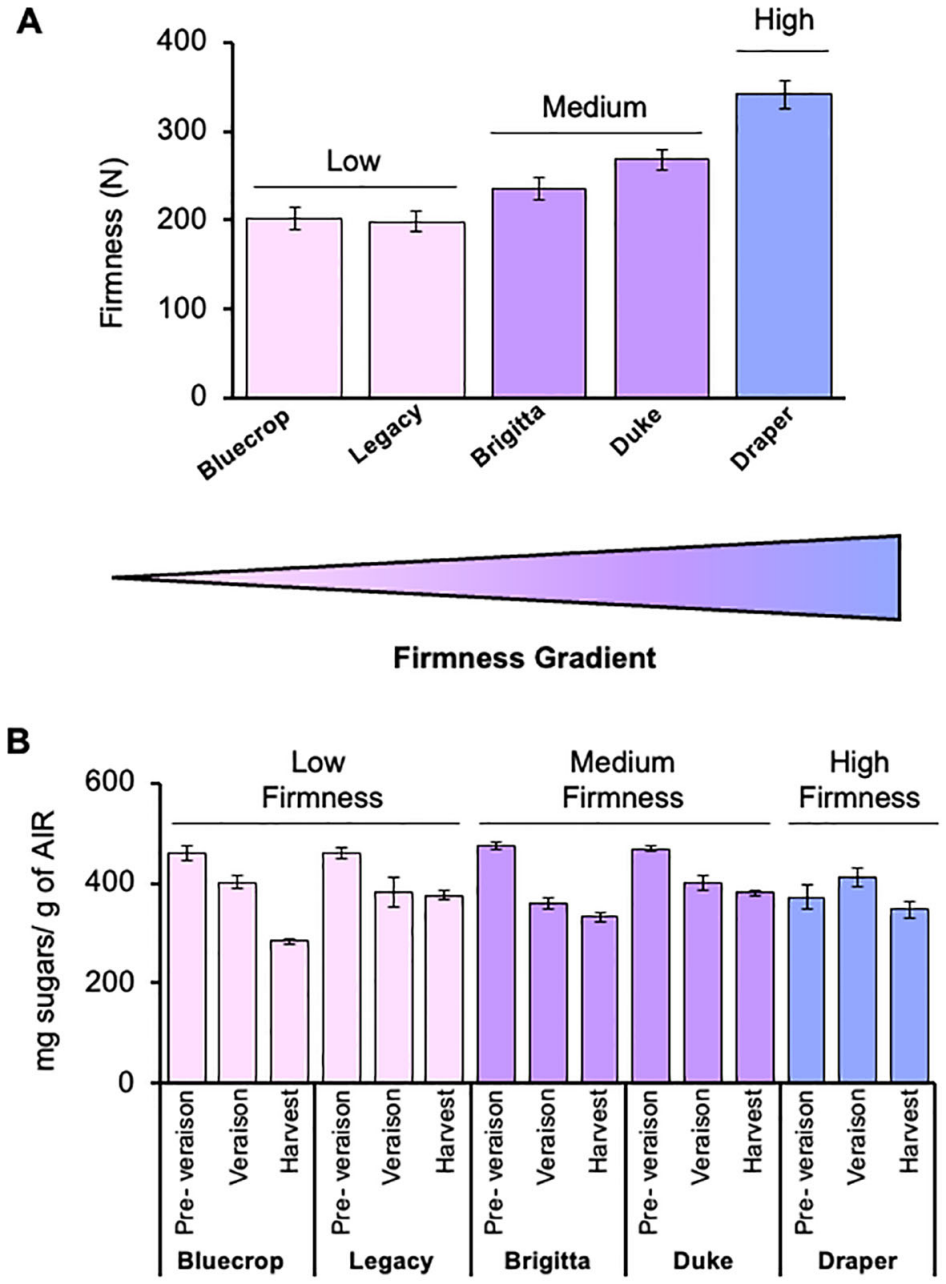
Total sugar content in five blueberry varieties with different firmness levels. **(A)** Firmness of five selected blueberry cultivars at the harvest stages. Bluecrop and Legacy belong to the low-firmness group, Brigitta and Duke are part of the intermediate-firmness group, while Draper is the firmest variety. Error bars represent the standard error (SE) from three biological replicates (*n* = 10). **(B)** Total monosaccharide content in the AIR fraction of the five blueberry varieties. AIR was hydrolyzed with TFA, and total sugar contents were determined by HPAEC-PAD. Error bars represent the standard error (SE) from three biological replicates (*n* = 12–18).

Firmness was evaluated in blueberries from Bluecrop, Legacy, Brigitta, Duke, and Draper varieties at the commercial harvest stage (**Figure 1A**). The firmness values of these varieties ranged from 198.6 to 341.3 N, with Draper being the firmest and Bluecrop and Legacy the softest (**Figure 1A**). The cultivars were categorized into three firmness groups: low (Bluecrop and Legacy, 202.4 and 198.6 N, respectively), medium (Brigitta and Duke, 234.9 and 268.5 N, respectively), and high (Draper, 341.3 N).

The total monosaccharide composition at each developmental stage was analyzed using the whole AIR, allowing for comparisons among the three firmness groups from pre-veraison to harvest stages (**Figure 1B**). In the pre-harvest stages, the low- and medium-firmness groups exhibited similar sugar amounts, ranging from 475.2 to 459.9 mg of sugars per gram of AIR (**Figure 1B**; **Table 1**). Notably, the firmer Draper variety had a lower sugar content compared to softer varieties at the pre-veraison stage. As the fruit ripened, the soft- and medium-firmness groups showed significant decreases in total sugar content, with reductions of 38.4%, 18.4%, 30.7%, and 18% in Bluecrop, Legacy, Brigitta, and Duke, respectively, when comparing the pre-veraison and harvest stages. Draper, however, exhibited only a slight reduction (6.9%) in total sugar content, which was not statistically significant (**Figure 1B**). In fact, Draper is the only variety whose sugar content increased from pre-veraison to veraison, resulting in nearly constant sugar levels during development.

**Table 1:** Monosaccharide composition in AIR of the five blueberry varieties from pre-veraison until harvest.

Low-firmness varieties						
Variety	Bluecrop			Legacy		
Sugars	Pre-veraison	Veraison	Harvest	Pre-veraison	Veraison	Harvest
**Fuc**	1.48 (0.14)a	1.07 (0.08)b	0.91 (0.08)b	1.61 (0.06)a	1.17 (0.11)b	1.16 (0.06)b
**Rha**	6.21 (0.23)a	5.68 (0.18)ab	4.61 (0.35)b	6.47 (0.20)a	5.37 (0.34)b	5.57 (0.20)b
**Ara**	63.07 (4.75)a	55.23 (4.10)ab	42.00 (3.20)b	64.17 (1.85)a	58.68 (5.97)b	49.30 (1.85)b
**Gal**	39.93 (2.81)	47.55 (3.38)	44.92 (3.12)	42.84 (1.008)b	46.43 (4.60)b	55.47 (1.08)a
**Glc**	22.96 (1.23)a	18.31 (1.10)a	12.38 (0.60)b	25.10 (1.08)a	18.74 (2.16)b	19.62 (0.60)ab
**Man**	6.79 (0.59)a	8.32 (0.65)a	6.28 (0.33)a	7.77 (0.32)ab	6.43 (0.65)b	9.94 (0.32)a
**Xyl**	231.54 (6.78)a	166.11 (8.92)b	126.90 (6.42)b	201.86 (7.06)a	135.19 (8.17)b	117.80 (7.06)b
**GalA**	93.19 (7.68)a	99.29 (8.75)a	109.55 (5.55)a	95.36 (3.18)b	102.08 (11.50)ab	116.55 (2.7)a
**Total sugars**	459.91 (13.82)	401.55 (14.23)	283.15 (6.28)	460.06 (9.95)	380.91 (29.85)	375.42 (9.97)
Medium-firmness varieties						
Variety	Brigitta			Duke		
Sugars	Pre-veraison	Veraison	Harvest	Pre-veraison	Veraison	Harvest
**Fuc**	1.30 (0.07)a	0.94 (0.07)b	0.95 (0.04)b	1.54 (0.11)a	1.16 (0.08)ab	1.21 (0.04)b
**Rha**	6.71 (0.36)a	5.29 (0.34)b	5.25 (0.15)b	7.19 (0.35)a	6.13 (0.26)b	6.60 (0.12)ab
**Ara**	85.27 (3.67)a	53.51 (2.37)b	51.04 (1.82)b	74.05 (4.71)a	56.57 (3.33)ab	46.04 (0.83)b
**Gal**	55.26 (2.44)	47.75 (2.14)	56.20 (2.01)	53.70 (1.96)b	49.14 (2.65)b	69.25 (1.61)a
**Glc**	38.17 (1.83)a	21.37 (1.01)b	20.78 (0.68)b	33.92 (1.25)a	22.51 (1.23)b	23.90 (0.67)b
**Man**	11.28 (1.03)a	8.86 (0.69)a	11.90 (0.83)a	11.85 (0.82)a	10.02 (0.90)a	12.20 (1.01)a
**Xyl**	104.93 (3.16)a	82.63 (2.77)b	57.15 (3.36)c	120.60 (6.71)a	133.51 (11.45)a	76.53 (7.33)b
**GalA**	157.03 (3.63)a	121.13 (5.25)b	132.40 (4.80)b	130.96 (8.09)a	120.91 (10.75)a	146.95 (3.76)a
**Total sugars**	475.25 (8.12)	359.45 (10.52)	331.82 (10.19)	468.46 (5.50)	399.95 (16.51)	380.86 (5.10)
High-firmness variety						
Variety	Draper					
Sugars	Pre-veraison	Veraison	Harvest			
**Fuc**	1.40 (0.11)a	1.07 (0.08)ab	0.98 (0.06)b			
**Rha**	6.24 (0.42)a	5.63 (0.29)a	5.21 (0.25)a			
**Ara**	78.91 (5.94)a	55.96 (4.78)b	51.37 (3.14)b			
**Gal**	39.85 (2.71)a	39.62 (3.17)a	44.13 (2.29)a			
**Glc**	33.65 (2.03)a	21.85 (1.98)b	19.21 (0.98)b			
**Man**	10.65 (0.68)a	8.76 (1.00)a	10.33 (0.51)a			
**Xyl**	95.30 (5.31)a	121.19 (16.13)a	92.90 (11.50)a			
**GalA**	124.00 (8.30)a	112.31 (11.98)a	110.16 (7.48)a			
**Total sugars**	372.02 (24.03)	410.76 (18.11)	346.36 (16.52)			

Sugar contents were determined for AIR fractions. Values represent the means and SE (in parentheses) of three biological repeats (n = 12–18). Letters denote differences between different monosaccharides for each variety, with statistical analyses performed using the Tukey test. Data were compared between each variety with p < 0.0001.

### 3.2 Monosaccharide analysis of AIR during blueberry ripening highlights varietal changes in xylose content

**Figure 2:**
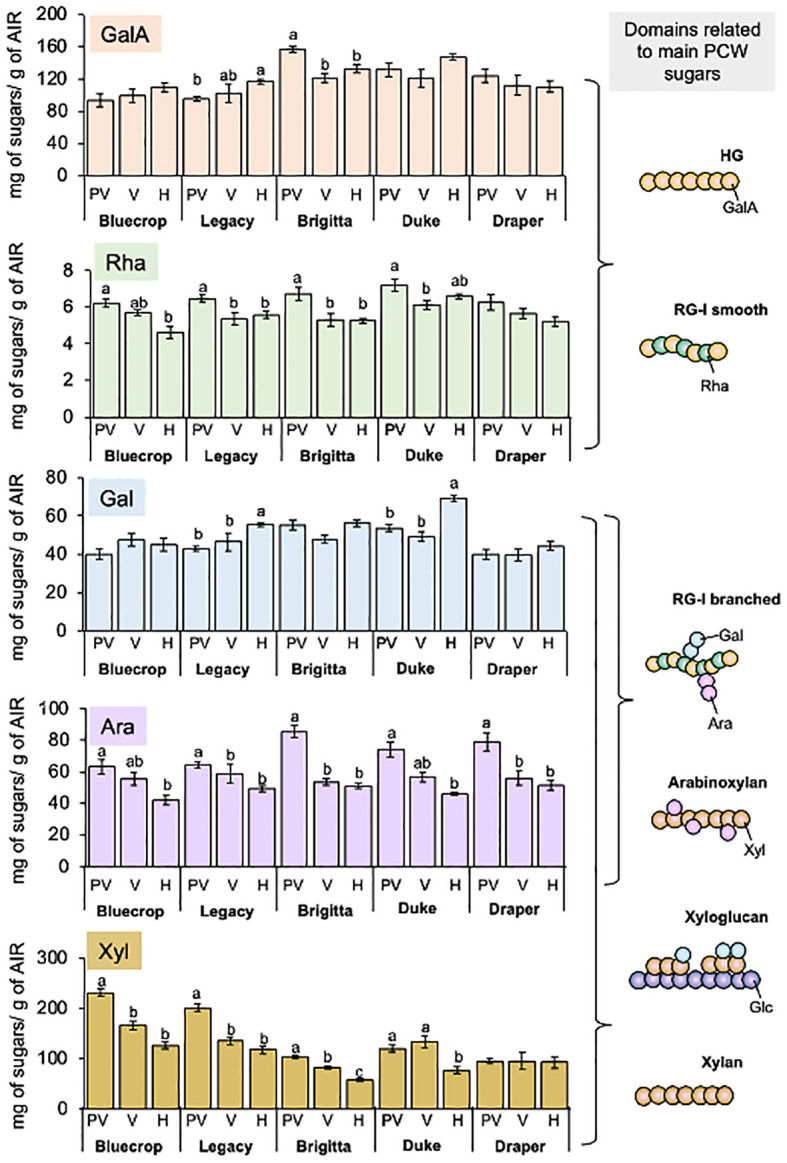
Main monosaccharide composition in the AIR fraction of examined blueberry varieties. The AIR was subjected to TFA hydrolysis, and the monosaccharide content was subsequently quantified using HPAEC-PAD. The graphs depict the content of GalA, Rha, Gal, Ara, and Xyl. Error bars represent the standard error (SE) from three biological replicates (*n* = 12–18). On the right side, schematic representations of the principal pectic and hemicellulose domains are presented, offering insights into the specific sugar and its corresponding domain. Significant alterations were observed in the sugars comprising the HG, RG-I, and HC polysaccharides, suggesting structural modifications within these polysaccharides. The absence of letters indicates no statistical differences. PV, Pre-Veraison; V, Veraison; H, Harvest.

To understand the changes in total sugars composing AIRs in the five varieties, a detailed monosaccharide composition was conducted from pre-veraison to harvest (**Table 1** and **Figure 2**). **Figure 2** illustrates the main sugar components in the five blueberry varieties: GalA, Rha, Gal, Ara, and Xyl, which are consistent with data previously reported on blueberries (Blaker and Olmstead, 2014; Chea et al., 2019; Lin et al., 2019; Trandel-Hayse et al., 2023), highlighting the variations in each phenotype group during fruit ripening. The GalA content remained relatively stable during fruit development, except for Legacy and Brigitta, which exhibited an increase and a decrease, respectively (**Figure 2**). Rhamnose content showed more significant changes, especially in the softer varieties like Bluecrop, Legacy, and Brigitta, where a substantial decrease occurred during ripening. This trend was less pronounced in Duke and Draper, which showed only a mild, statistically significant decrease. The Gal content remained almost constant for most varieties, showing only a mild increase throughout ripening. However, Legacy and Duke exhibited a significant increase from pre-veraison to harvest. Notably, Draper, the firmer variety, consistently maintained the lowest Gal content at all stages compared to the softer varieties. A significant decline in Ara content was observed in all varieties during the ripening process. In contrast, variations in Xyl content highlighted a distinct profile between Draper, the firmer variety, and the less firm ones. Bluecrop, Legacy, Brigitta, and Duke all showed a significant reduction in Xyl content from pre-veraison to harvest, while Draper maintained a relatively constant level of Xyl throughout development.

**Table 1** provides additional details on minor sugars, including Glc, Man, and Fuc. Across all varieties, a decrease in Fuc and Glc content was observed during fruit ripening, while no significant changes were noted for Man content.

### 3.3 The alteration in the degree of methylesterification during blueberry development plays a role in preserving fruit firmness

**Figure 3:**
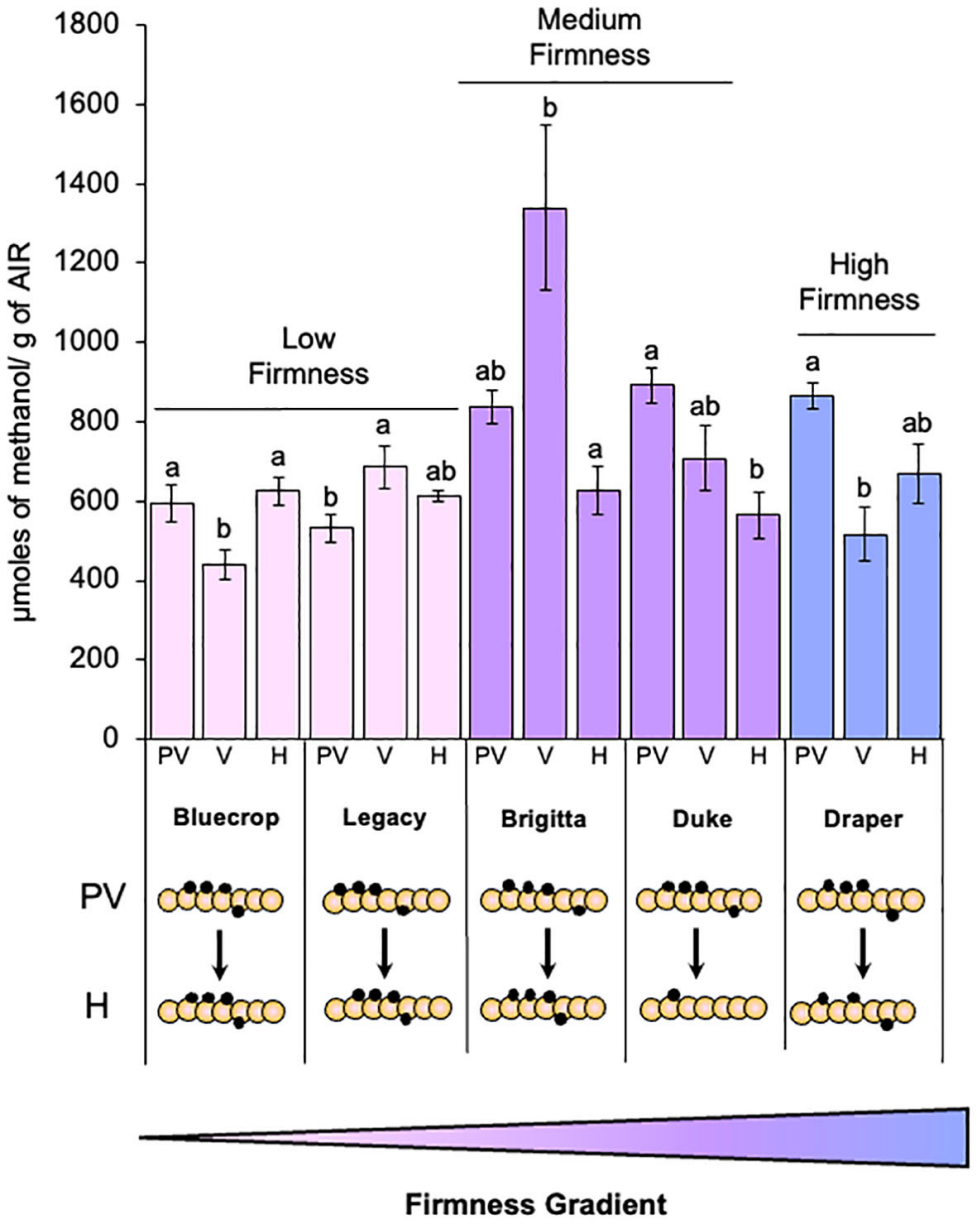
Methanol content in the AIR fractions of the studied blueberry varieties. The quantification of methanol content was carried out following saponification of the AIR with NaOH. Results are expressed as micromoles of methanol per gram of AIR. Error bars represent the standard error (SE) with *n* = 15, derived from three biological replicates. Letters indicate differences among the different blueberry varieties, with statistical analysis performed using the Tukey test with a significance level of 0.01. PV, Pre-Veraison; V, Veraison; H, Harvest. Schematic representations of HG domains are presented, providing insights into changes in the methylation domains. GalA (orange circles), methyl groups (black circles).

The relationship between fruit firmness and cell wall stiffening, mediated by calcium cross-linking of the HG domain, has been extensively documented. This process is closely associated with the presence and distribution pattern of methyl groups in the GalA residues that constitute the HG. Therefore, we investigated the relationship between pectin methylesterification and berry firmness phenotype by quantifying methanol content in AIR for all varieties at three different developmental stages (**Figure 3**).

Interestingly, a correlation between methanol content profile and fruit firmness increase was observed. In low-firmness fruit, methanol content showed minimal variation during fruit development, with only a slight increase observed at harvest compared to pre-veraison. Conversely, medium- and high-firmness berries displayed a consistent decrease in methanol content, with all three varieties showing lower levels at harvest than at pre-veraison. These findings suggest an association between the methylesterification of cell wall polysaccharides and blueberry firmness, indicating that varieties displaying a decline in methanol content during development tend to have higher firmness values (**Figure 3**).

### 3.4 Analysis of pectin-enriched fractions and cytological analysis confirm the relevance of HG methyl esterification in blueberry firmness

**Figure 4:**
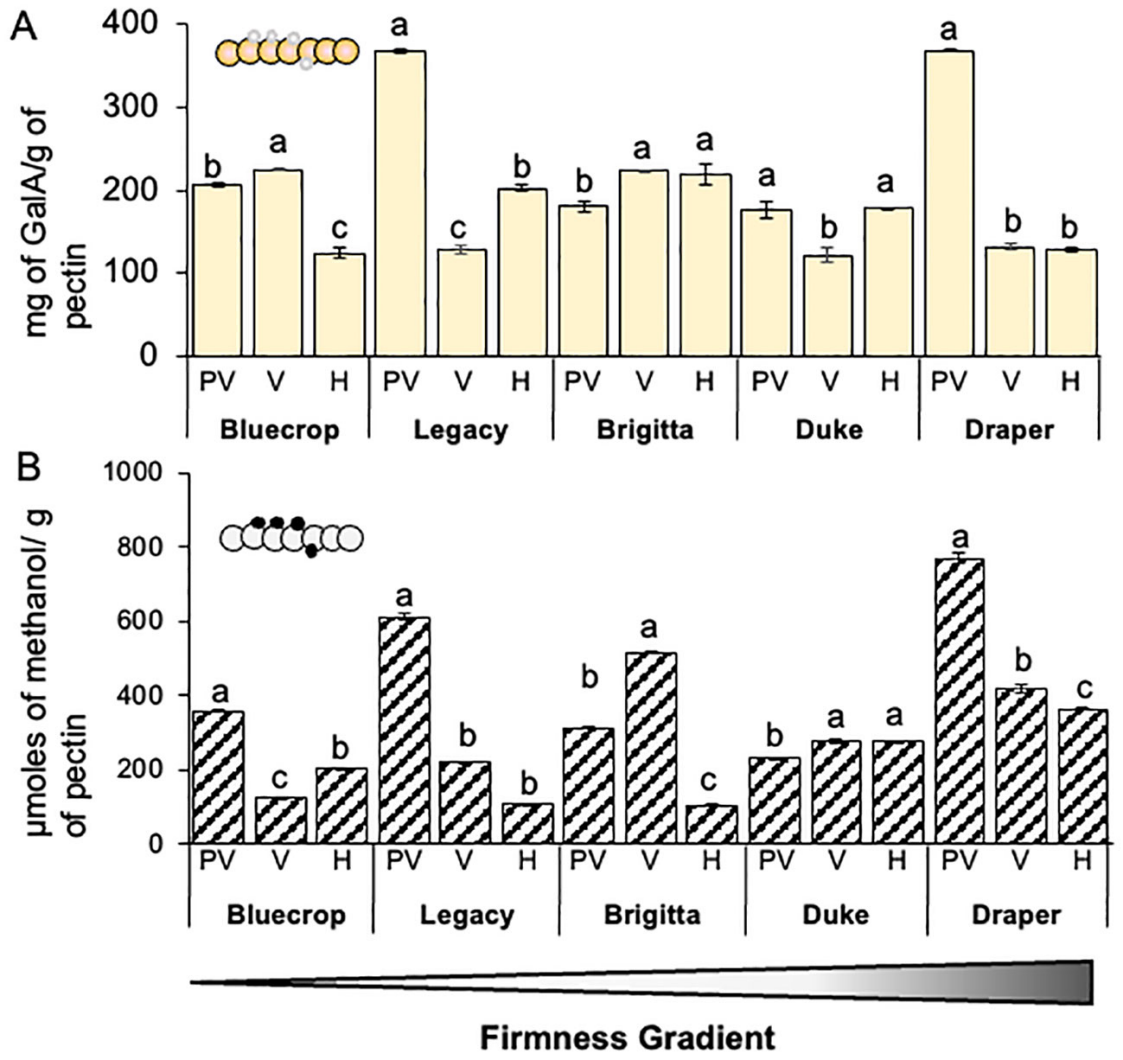
Demethylesterification of homogalacturonan domains in pectins is more pronounced in firmer varieties. **(A)** Quantification of galacturonic acid (GalA) in pectin fractions. The pectin fraction of blueberry varieties underwent hydrolysis with TFA, and the GalA content was determined using HPAEC-PAD. Error bars represent the standard error (SE) from three technical replicates (*n* = 3). One-way ANOVA tests were conducted, and data were compared between each variety with *p* < 0.0001. **(B)** Measurement of methanol content in blueberry varieties after pectin saponification with NaOH using a colorimetric assay. Error bars denote the standard error (SE) with *n* = 5. One-way ANOVA and Tukey tests were performed, comparing data between each variety with *p* < 0.0001. PV, Pre-Veraison; V, Veraison; H, Harvest. Schematic representations of homogalacturonan domains are presented in the top of each graph. Schematic representations of HG are presented, providing insights into structural changes in these domains. GalA (large circles), methyl groups (small circles).

The hydrolysis of AIR is complex due to the entangled polysaccharide network that makes up the PCW. To disentangle this complex polymer network and avoid any bias in monosaccharide quantification, we separated pectins from the AIR and analyzed their composition using HPAEC-PAD and colorimetric assays (**Figure 4A** and **Table 2**). A decrease in GalA content was observed in both softer and firmer varieties (Bluecrop, Legacy, and Draper), whereas the medium-firmness varieties Brigitta and Duke showed a slight increase or maintained GalA content during fruit ripening.

**Table 2:** Monosaccharide composition of the enriched pectin fraction from the five blueberry varieties from pre-veraison until harvest.

Low-firmness varieties						
Variety	Bluecrop			Legacy		
Sugars	Pre-veraison	Veraison	Harvest	Pre-veraison	Veraison	Harvest
**Fuc**	N.d	N.d	N.d	N.d	N.d	N.d
**Rha**	2.13 (0.05)c	3.84 (0.09)a	3.12 (0.07)b	3.11 (0.05)b	1.52 (0.00)c	3.44 (0.09)a
**Ara**	35.36 (0.06)c	94.59 (0.84)a	39.14 (0.55)b	50.07 (0.75)a	35.29 (0.25)c	45.01 (0.85)b
**Gal**	15.99 (0.10)c	36.25 (0.51)a	22.41 (0.14)b	19.20 (0.25)b	14.89 (0.11)c	25.02 (1.16)a
**Glc**	10.40 (0.20)b	18.23 (0.47)a	6.70 (0.05)c	14.38 (0.24)a	10.15 (0.11)b	7.04 (0.16)c
**Man**	1.44 (0.02)a	1.30 (0.07)a	1.30 (0.03)a	1.19 (0.06)a	0.34 (0.01)b	1.36 (0.17)a
**Xyl**	12.50 (0.04)b	17.98 (0.09)a	7.81 (0.47)c	26.78 (0.54)a	10.41 (0.11)b	7.42 (0.27)c
**GalA**	207.00 (2.14)b	225.22 (1.68)a	124.70 (6.03)c	369.09 (2.41)a	128.78 (4.52)c	202.23 (3.83)b
**GlcA**	N.d	N.d	N.d	N.d	N.d	N.d
**Total sugars**	284.82 (2.41)	397.42 (1.53)	205.19 (5.84)	483.83 (4.01)	201.38 (4.45)	291.53 (5.41)
Medium-firmness varieties						
Variety	Brigitta			Duke		
Sugars	Pre-veraison	Veraison	Harvest	Pre-veraison	Veraison	Harvest
**Fuc**	N.d	N.d	N.d	N.d	N.d	N.d
**Rha**	1.71 (0.02)c	1.97 (0.07)b	2.77 (0.07)a	1.20 (0.08)b	1.37 (0.12)b	2.79 (0.02)a
**Ara**	28.09 (0.18)b	32.85 (0.31)a	31.20 (0.78)a	21.06 (1.69)b	14.51 (1.05)c	31.01 (0.08)a
**Gal**	11.92 (0.11)c	18.16 (0.16)b	20.94 (0.40)a	11.23 (0.96)b	7.69 (0.60)c	28.85 (0.04)a
**Glc**	8.69 (0.08)b	9.65 (0.30)a	6.05 (0.11)c	6.12 (0.50)b	4.49 (0.36)c	9.24 (0.06)a
**Man**	1.06 (0.07)a	0.58 (0.06)b	1.19 (0.04)a	1.43 (0.17)a	0.52 (0.11)b	1.18 (0.01)a
**Xyl**	5.41 (0.05)c	8.84 (0.05)a	7.00 (0.12)b	6.36 (0.57)b	4.01 (0.53)c	12.82 (0.04)a
**GalA**	181.02 (5.95)b	223.19 (2.18)a	219.12 (12.94)a	176.44 (10.90)a	121.95 (8.24)b	179.67 (0.51)a
**GlcA**	N.d	N.d	N.d	N.d	N.d	N.d
**Total sugars**	237.9 (6.04)	295.25 (1.99)	288.27 (14.03)	223.84 (14.85)	154.54 (10.98)	265.56 (0.69)
High-firmness varieties						
Variety	Draper					
Sugars	Pre-veraison	Veraison	Harvest			
**Fuc**	N.d	N.d	N.d			
**Rha**	1.76 (0.07)b	1.17 (0.02)c	3.08 (0.07)a			
**Ara**	43.94 (0.10)a	19.98 (0.45)b	16.52 (0.06)c			
**Gal**	10.64 (0.13)a	5.91 (0.26)c	8.22 (0.05)b			
**Glc**	13.61 (0.06)a	5.99 (0.33)b	4.68 (0.08)c			
**Man**	1.56 (0.03)a	0.51 (0.10)b	0.51 (0.09)b			
**Xyl**	12.91 (0.06)a	6.00 (0.24)b	3.80 (0.13)c			
**GalA**	368.77 (0.99)a	131.88 (4.34)b	129.50 (2.63)b			
**GlcA**	N.d	N.d	N.d			
**Total sugars**	453.19 (0.90)	171.44 (3.05)	166.31 (2.47)			

Sugar contents were determined for pectin fractions. Values represent the means and SE (in parentheses) of three technical repeats (n = 3). Letters denote differences between different monosaccharides for each variety, with statistical analyses performed using the Tukey test. Data were compared between each variety with p < 0.0001. N.d, Not determined.

The pectin-enriched fractions were analyzed for their methanol content (**Figure 4B**). All varieties, except Duke, which remained fairly stable, showed a significant decrease in methanol content from pre-veraison to harvest. The most substantial decreases were observed in the Legacy and Draper varieties.

**Figure 5:**
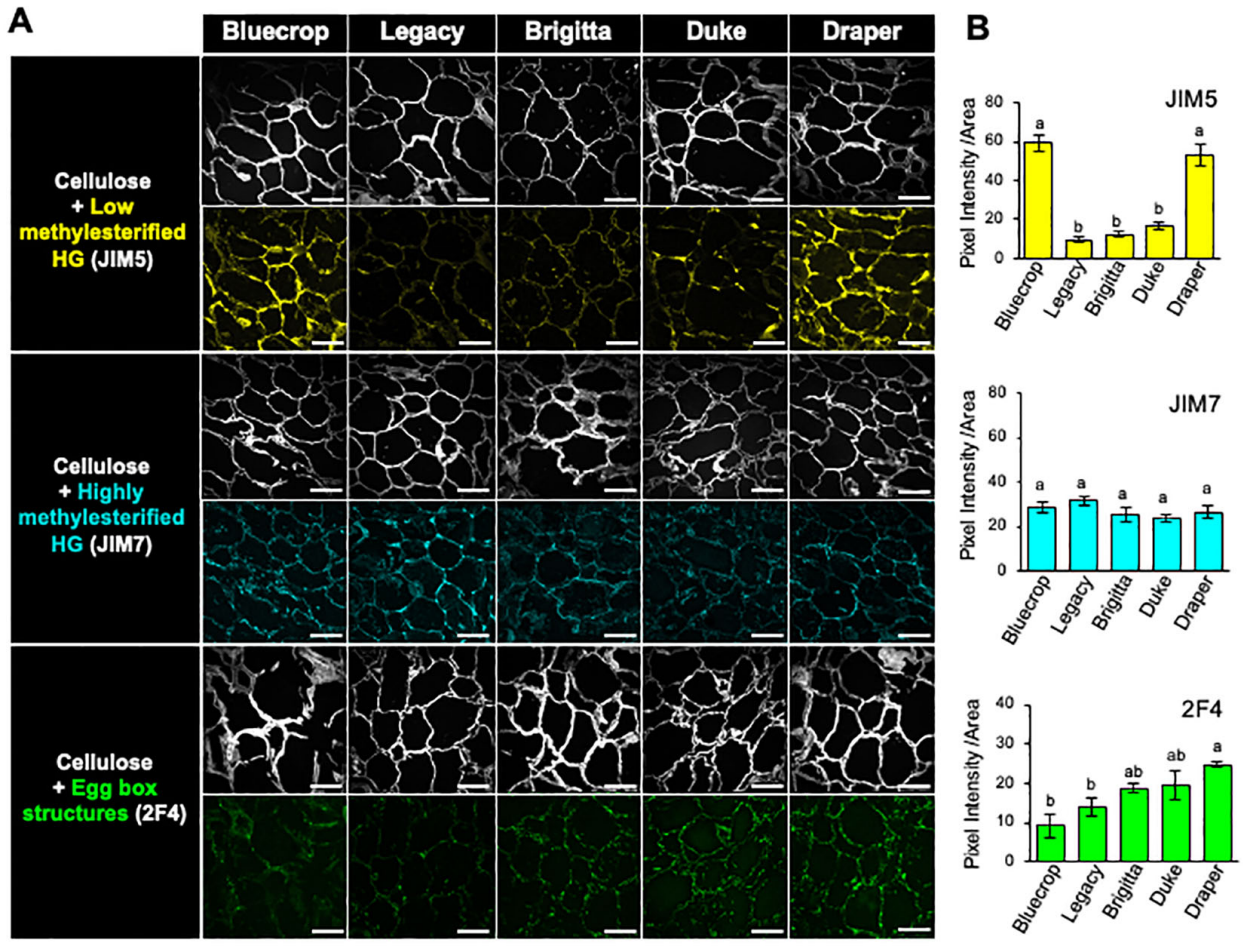
Immunolabeling of HG epitopes at the harvest stage of fruit sections in the five blueberries, revealing enhanced accumulation of demethylesterified HG and egg-box structures in firmer fruit cell wall. **(A)** Optical sections of mature blueberries were obtained by confocal microscopy. Fluorescent signals corresponding to JIM5, JIM7, and 2F4 antibodies were used to label low methylesterified HG (in yellow), highly methylesterified HG (in cyan), and egg-box structures (in green), respectively. Simultaneously, calcofluor white, detecting beta-1,4-glucans, was used and visualized in gray. Scale bar = 100 μm. **(B)** Quantification of HG antibody labeling shown in **(A)**. Colored pixels on the sections were quantified from over three images for each variety. The values represent the means, and the standard error (SE) was calculated from three biological replicates. Letters indicate differences between the different blueberry varieties, with statistical analysis performed using the Tukey test with a significance level of 0.01.

Another important factor in cell wall stiffening is the consideration of both the methanol content and the epitope distribution within the cell wall. To assess this, we analyzed the distribution of HG within the cell wall through immunolabeling of HG epitopes in fruit sections at the harvest stage (**Figure 5**). Specific antibodies were used for labeling low methylesterified HG (JIM5), highly methylesterified HG (JIM7), and egg-box structures formed by demethylesterified HG (2F4) (Vandenbosch et al., 1989; Knox et al., 1990; Liners et al., 1989). Simultaneously, cellulose was stained with calcofluor white to visualize the cell wall structure (**Figure 5A**). The cellulose staining revealed heterogeneous forms of the flesh cells while maintaining their integrity. In Bluecrop and Draper varieties, JIM5 showed strong labeling, whereas it exhibited lower labeling in Legacy, Brigitta, and Duke (**Figure 5A**). Conversely, JIM7 displayed a consistent pattern of epitope accumulation and intensity across all five varieties (**Figure 5B**). The detection of egg-box structures indicated increased epitope accumulation in the firmer varieties, as confirmed by pixel intensity measurements (**Figures 5A, B**).

### 3.5 The analysis of rhamnogalacturonan-I highlighted its significance and emphasized the importance of arabinogalactan proteins in blueberry firmness

**Figure 6:**
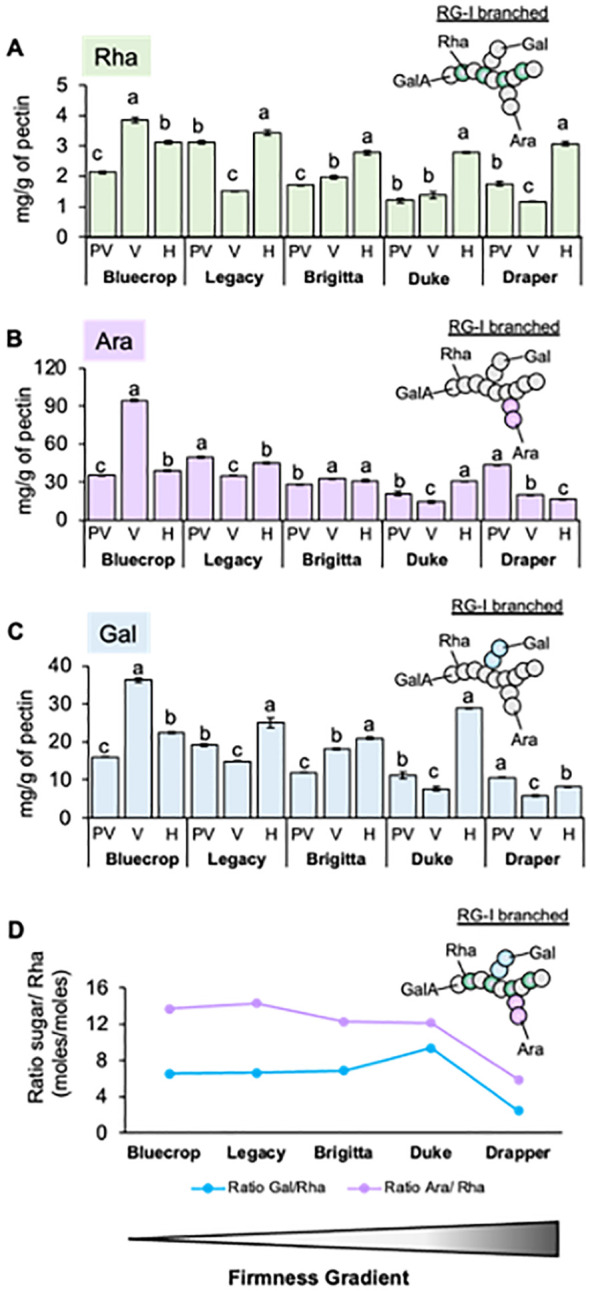
Variation in rhamnogalacturonan-I structure in firmer blueberry varieties. The pectin fraction was extracted from the AIR of the five blueberry varieties by separating it from cellulose and hemicellulose using different chemical eluants. **(A–C)** Content of Rhamnose [Rha, **(A)**], Arabinose [Ara, **(B)**], and Galactose [Gal, **(C)**] sugars in the pectin fraction of the five blueberry varieties during ripening. The pectin-purified fraction was hydrolyzed with TFA, and the content of monosaccharides was determined using HPAEC. Error bars represent the standard error (SE) from three technical replicates (*n* = 3). **(D)** Ratio between the number of Ara, Gal, and Rha moles in the pectin fraction at the harvest stage. The values represent the means, and the error bars represent the SE values from four biological replicates (*n* = 4). Differences observed for the Ara/Rha and Gal/Rha ratios in Draper suggest changes in the RG-I structure that might relate to firmness. PV, Pre-Veraison; V, Veraison; H, Harvest. Schematic representations of RG-I are presented, providing insights into structural changes in these domains.

In addition to changes in GalA, the amounts and variations of Rha, Ara, and Gal sugars in the pectin-enriched fraction of blueberry varieties are shown in **Figure 6**. These three monosaccharides are the main RG-I components, suggesting that subtle changes in this domain could affect fruit firmness. During fruit ripening, the Rha content increases across all varieties (**Figure 6A**). A slight increase in both Ara and Gal content is observed in all the varieties during ripening, except for Draper, which is the firmer variety (**Figures 6B, C**). The Ara/Rha and Gal/Rha ratios are similar for most varieties, except for Draper, whose ratios are smaller, indicating a higher proportion of Rha in the firmer variety (**Figure 6D**). These findings suggest that there is less RG-I branching in Draper.

**Figure 7:**
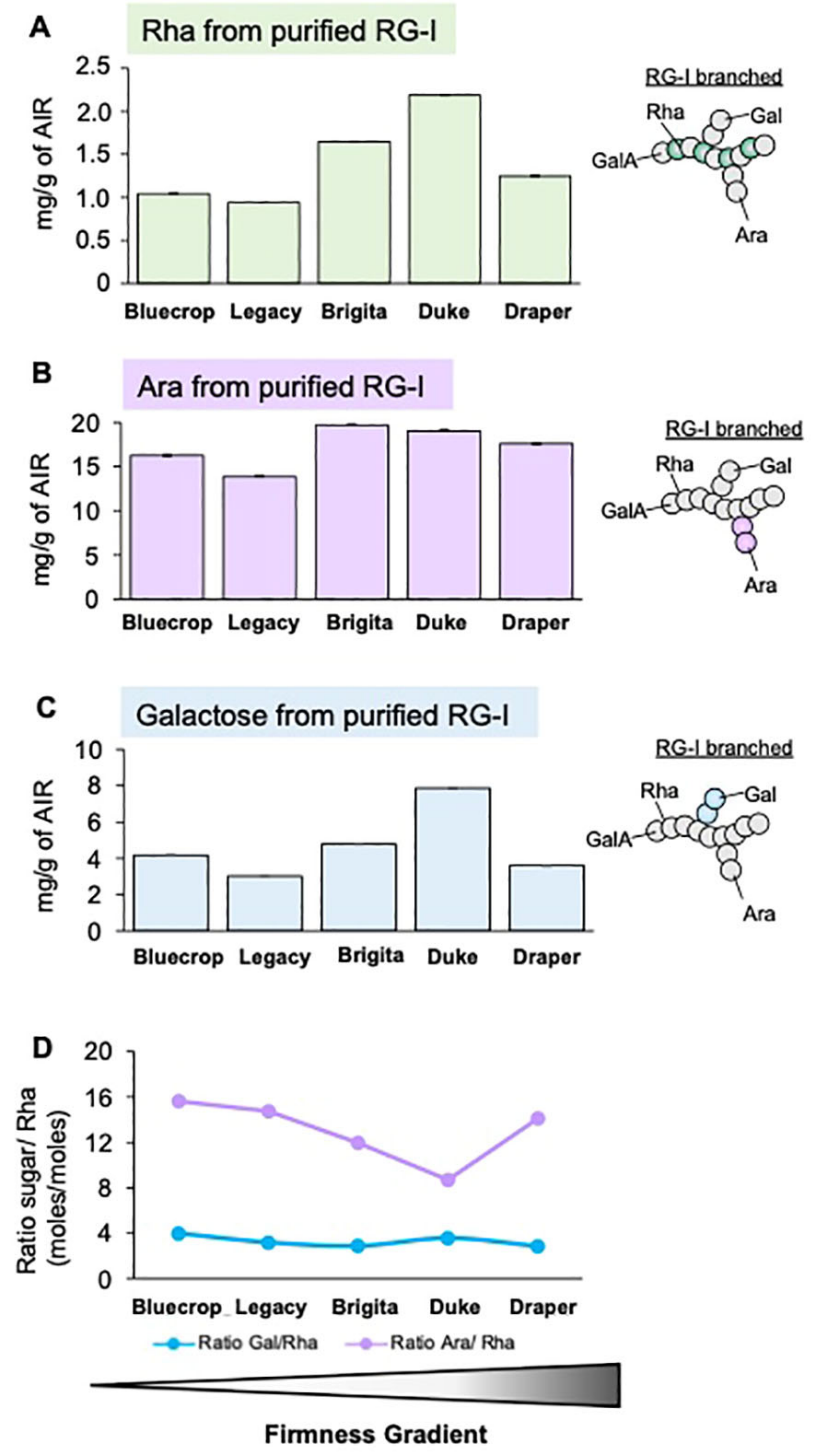
Comparison of purified RG-I sugar composition reveals minimal differences among blueberry varieties at the harvest stage. The RG-I domain was isolated from the five blueberry varieties at the harvest stage, following AIR digestion with endopolygalacturonase and size separation by a BioGel P-30 column. Collected RG-I fractions were pooled before hydrolyzed with TFA, and monosaccharide contents were determined by HPAEC-PAD. **(A–C)** Rhamnose [Rha, **(A)**], Arabinose [Ara, **(B)**], and Galactose [Gal, **(C)**] sugar composition of the purified RG-I fraction. Error bars represent the SE from three technical replicates (*n* = 3). **(D)** Ratio between the number of Ara or Gal moles to Rha moles in RG-I fraction at the harvest stage. Values represent the means, and error bars represent SE values from three technical replicates (*n* = 3). Schematic representations of RG-I are presented, providing insights into structural changes in these domains.

In the pectin-enriched fraction, neutral sugars such as Glc, Man, and Xyl, typically found in HCs, were also detected (**Table 2**), suggesting contamination of the pectic fraction with hemicellulosic components. To prevent contamination, the RG-I domain at the harvest stage was isolated through endo-PG digestion and gel filtration chromatography. After isolation, the RG-I fractions were analyzed by HPAEC-PAD to quantify their monosaccharide composition. The results presented in **Figure 7** show no distinct pattern in the levels of Rha, Ara, and Gal in the isolated RG-I (**Figures 7A–C**) relative to fruit firmness. However, examining the ratios of Ara/Rha and Gal/Rha (**Figure 7D**) reveals that the Gal/Rha ratio tends to remain stable across all varieties, with only a subtle decrease in Draper. For the Ara/Rha ratio, we observe a decrease in the medium-firmness varieties. Draper is an exception, as its ratio is similar to that of the softer fruit.

**Figure 8:**
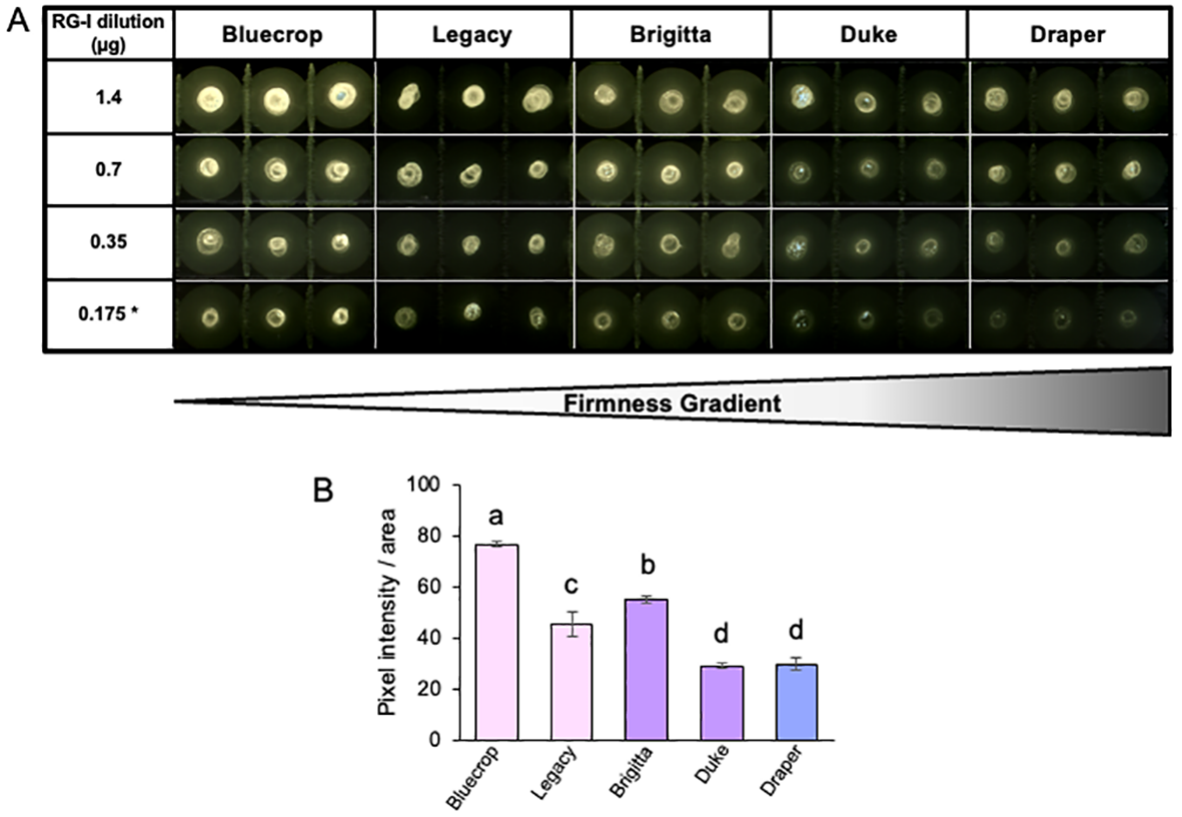
Decreased AGP epitopes detected on isolated RG-I in firmer blueberry fruit. **(A)** Dot blots were conducted on different dilutions of the purified RG-I fraction using the LM30 antibody that specifically targets arabinogalactan proteins (AGPs). Reduced intensity of AGP epitopes is observed in firmer blueberries. **(B)** Quantification of 0.7-µg dilution of LM30 dot blot. Letters indicate significant differences between varieties. The standard error (SE) was calculated from three technical replicates (*n* = 3). Student’s *t*-test comparison analyses were performed with *p* < 0.05.

**Figure 9:**
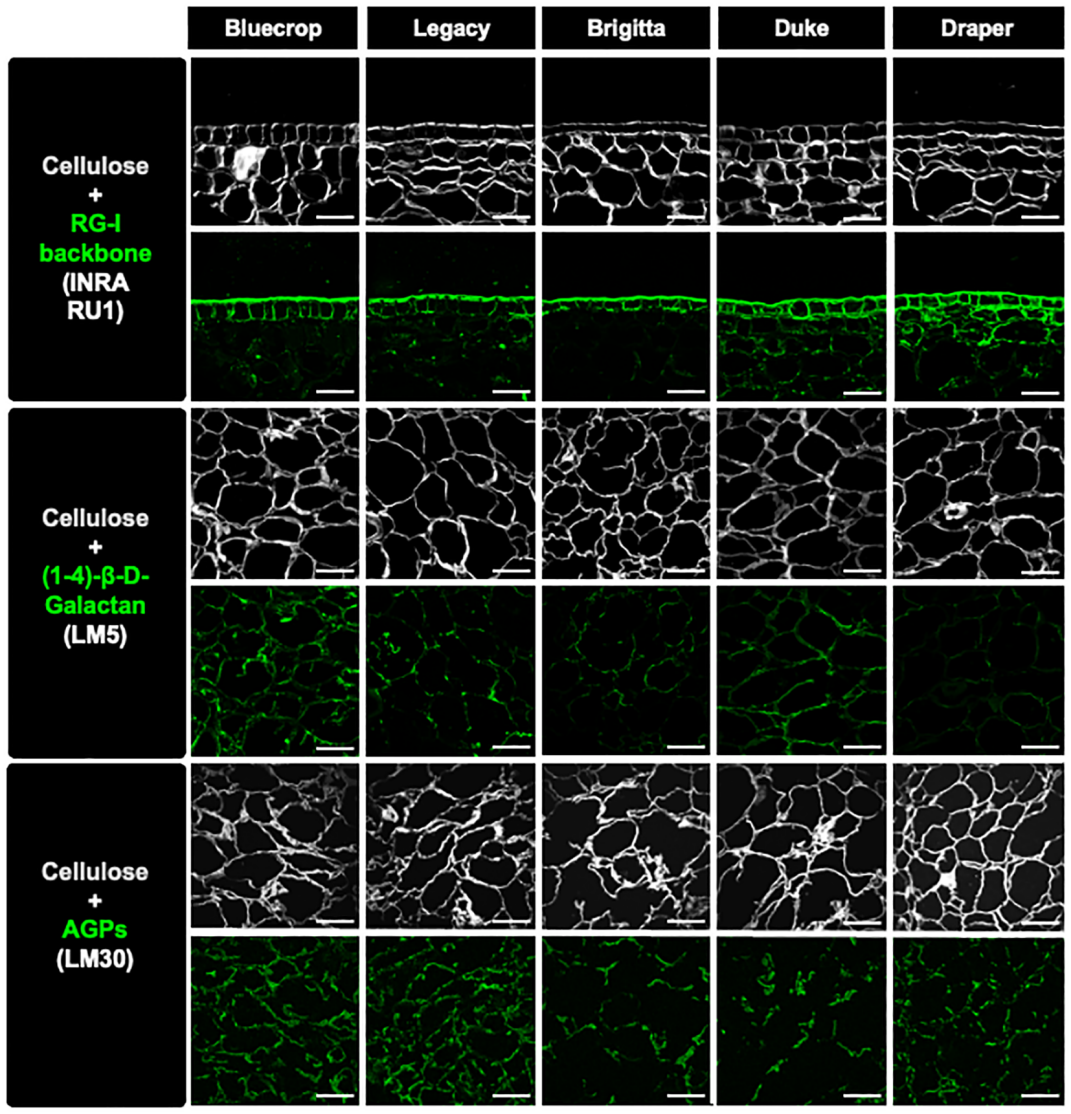
Immunolabeling analysis of fruit sections at the harvest stage reveals reduced RG-I ramification and AGP detection in the cell wall of firmer fruit. Sections from mature blueberries were imaged using confocal microscopy to reconstruct optical sections. Immunolabeling was performed using INRA-RU1, LM5, and LM30 antibodies to specifically label the RG-I backbone, galactan chains, and arabinogalactan proteins (AGPs), respectively. These labels were visualized in green fluorescence. Simultaneously, calcofluor white, which detects beta-1,4-glucans, was used and appeared as gray fluorescence. The scale bar represents 100 μm.

Recent evidence indicates that AGPs are linked to the RG-I domains (Tan et al., 2023a, 2024). To investigate potential changes in AGPs within the purified RG-I fraction, we conducted a dot blot assay using isolated RG-I fractions and the LM30 antibody, which targets AGPs (Moller et al., 2008) (**Figure 8A**). The results of the dot blot analysis demonstrate a substantial decrease in AGP detection specifically in the firmer varieties (**Figure 8B**), suggesting a potential involvement of AGPs in the softer phenotype. To provide *in vitro* evidence of changes in the RG-I structure in the firmer varieties, immunolabeling experiments were conducted on fruit sections at the harvest stage. Specific antibodies were used to target RG-I backbone (INRA-RU1), galactan side chains (LM5), and AGP epitopes (LM30) (Ralet et al., 2010; Jones et al., 1997; Moller et al., 2008). Simultaneously, cellulose was stained with calcofluor white to visualize the overall cell wall structure, revealing a heterogeneous form of the flesh cells while maintaining their integrity (**Figure 9**). The label for unbranched RG-I was localized in the epidermal cells of the berry fruit. In the firmer fruit, there was an increase in labeling with an expanded spread of the label into the flesh cells, indicating a higher presence of unbranched RG-I in the firmer fruit. The LM5 antibody showed the most labeling for Bluecrop, with decreasing intensity in Legacy, Brigitta, and Duke, and an almost complete loss in Draper (**Figure 9**), suggesting less branching of the RG-I backbone. The accumulation of AGP epitopes in the cell wall exhibited a decrease in Brigitta, Duke, and Draper varieties, corroborating the findings from the dot blot assays (**Figure 8**). This evidence collectively emphasizes the role of RG-I structure and AGPs in fruit firmness, suggesting that a higher abundance of unbranched RG-I and a lower level of AGPs appear to be associated with a firmer cell wall structure.

### 3.6 Hemicellulose-enriched fractions reveal changes in xylan, arabinoxylan, and xyloglucan structure during fruit ripening in the five blueberry varieties

**Figure 10:**
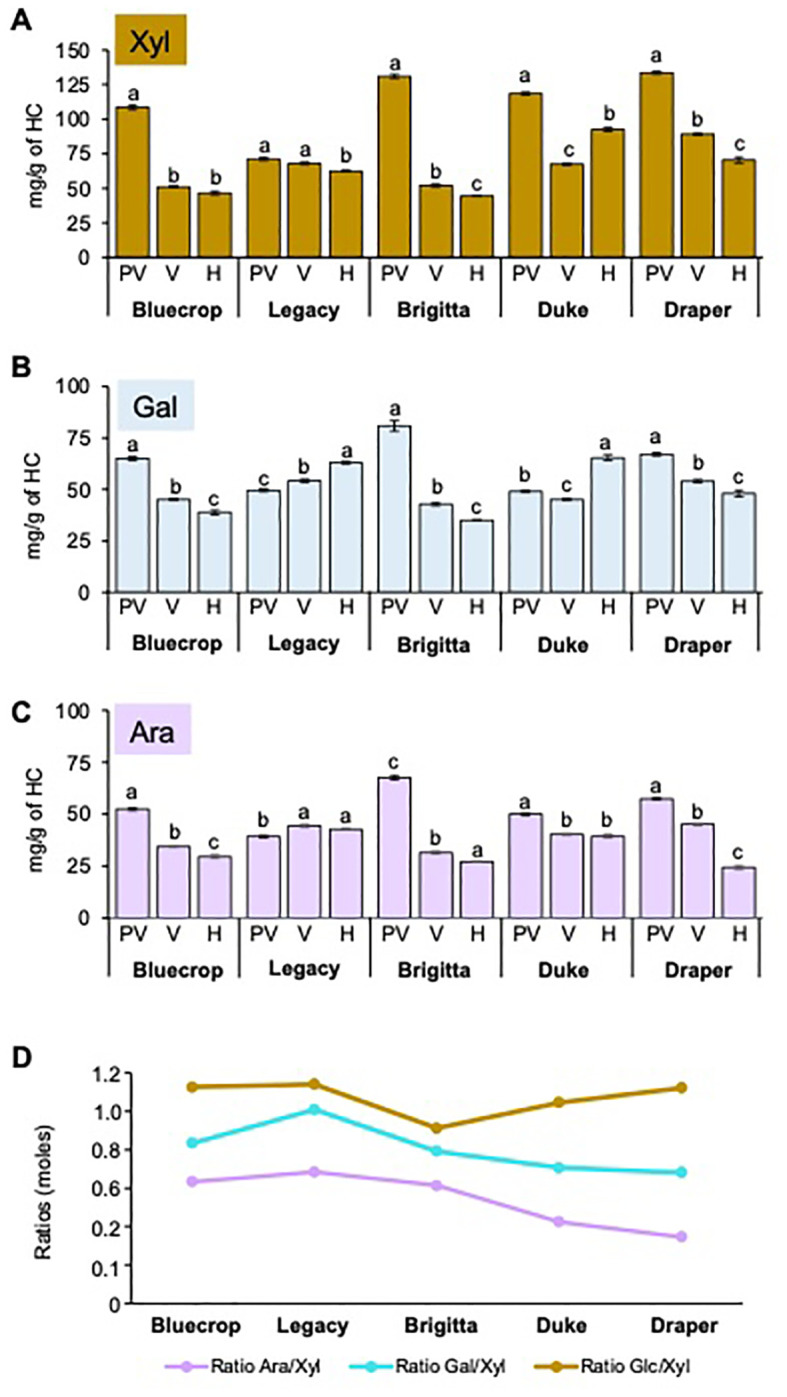
Comparison of purified hemicellulose sugar composition reveals changes between blueberry varieties during fruit ripening. HC from the five blueberry varieties were isolated, and the monosaccharide composition was determined by HPAEC-PAD after TFA hydrolysis. **(A–C)** Xylose [Xyl, **(A)**], Arabinose [Ara, **(B)**], and Galactose [Gal, **(C)**] in the HC fraction of the five blueberry varieties are represented. Error bars indicate the SE from three technical replicates (*n* = 3). One-way ANOVA and Tukey tests were performed, and data were compared between each variety with *p < 0.0001. **(D)** Ratio of Ara/Xyl and Xyl/Glc moles in the HC fraction at the harvest stage. The values represent the means, with error bars representing SE values from three technical replicates (*n* = 3). PV, Pre-Veraison; V, Veraison; H, Harvest.

An analysis of HC-enriched fractions was conducted using HPAEC-PAD, and the results are presented in **Table 3**. The primary sugars composing HC—namely, Xyl, Ara, and Gal—showed varying patterns (**Figure 10**). In the HC-enriched fractions, the Xyl content varied across the five varieties, with all showing a decrease during ripening across all varieties. Bluecrop, Brigitta, and Draper exhibited the most substantial reductions of 57.1%, 67%, and 47.2%, respectively. Despite the overall decrease in Xyl during fruit ripening, firmer fruit had higher Xyl content at harvest compared to the low-firmness varieties. Changes in Ara and Gal content were also noted, with decreases in Bluecrop, Brigitta, and Draper and increases in Legacy and Duke. Minor sugars such as Man and Fuc decreased in all varieties, except for Legacy, which maintained its Man content, and Duke, which showed an increase in Fuc. Finally, Glc, the second major sugar in HC, displayed varying behavior (**Table 3**). Two varieties showed only mild changes in Glc during softening, with an increase in Duke and a decrease in Legacy. In contrast, Bluecrop, Brigitta, and Draper exhibited significant decreases of 50.1%, 71.8%, and 46.4%, respectively, similar to the percentage values estimated for their Xyl decrease.

**Table 3:** Monosaccharide composition of the hemicellulose fraction from the five blueberry varieties from pre-veraison until harvest.

Low-firmness varieties						
Variety	Bluecrop			Legacy		
Sugars	Pre-veraison	Veraison	Harvest	Pre-veraison	Veraison	Harvest
**Fuc**	4.07 (0.08)a	1.66 (0.08)b	1.80 (0.06)b	2.81 (0.01)a	2.22 (0.16)b	2.09 (0.10)b
**Rha**	3.15 (0.04)a	2.19 (0.02)b	3.08 (0.07)a	2.25 (0.06)b	2.45 (0.02)ab	2.69 (0.07)a
**Ara**	52.45 (0.71)a	34.53 (0.22)b	29.60 (0.89)c	39.31 (0.62)b	44.39 (0.64)a	42.77 (0.25)a
**Gal**	65.05 (0.92)a	45.22 (0.41)b	38.91 (1.17)c	49.63 (0.66)c	54.24 (0.73)b	63.07 (0.61)a
**Glc**	107.34 (1.42)a	63.13 (0.80)b	52.54 (1.64)c	78.02 (1.19)a	75.64 (1.10)ab	71.24 (0.76)b
**Man**	25.51 (0.58)a	18.38 (1.48)b	12.36 (0.32)c	18.69 (0.20)b	20.29 (0.34)a	21.36 (0.32)a
**Xyl**	108.54 (1.79)a	51.00 (0.65)b	46.64 (1.32)b	71.25 (1.16)a	68.04 (0.87)a	62.48 (0.58)b
**GalA**	3.90 (0.07)b	4.64 (0.39)b	10.07 (0.29)a	3.04 (0.04)a	4.32 (0.70)a	4.36 (0.13)a
**GlcA**	N.d	N.d	N.d	N.d	N.d	N.d
**Total sugars**	370.02 (5.47)	220.76 (3.56)	194.99 (5.72)	264.99 (3.85)	271.6 (4.19)	270.06 (2.45)
Medium-firmness varieties						
Variety	Brigitta			Duke		
Sugars	Pre-veraison	Veraison	Harvest	Pre-veraison	Veraison	Harvest
**Fuc**	2.84 (0.13)a	1.05 (0.05)b	0.66 (0.06)c	1.93 (0.07)b	1.82 (0.02)b	2.28 (0.04)a
**Rha**	2.98 (0.15)a	1.42 (0.03)b	1.81 (0.02)b	3.63 (0.03)a	3.07 (0.09)b	3.06 (0.07)b
**Ara**	67.62 (1.10)c	31.63 (0.66)b	27.27 (0.08)a	50.01 (0.55)a	40.40 (0.38)b	39.45 (0.84)b
**Gal**	80.86 (2.52)a	42.94 (0.83)b	35.17 (0.03)c	49.22 (0.47)b	45.27 (0.47)c	65.48 (1.27)a
**Glc**	156.05 (2.56)a	62.29 (1.19)b	43.99 (0.04)c	90.20 (0.68)b	73.81 (1.07)c	96.79 (1.81)a
**Man**	30.63 (0.21)a	17.01 (0.22)b	11.91 (0.02)c	29.36 (0.27)a	18.95 (0.30)c	23.16 (0.50)b
**Xyl**	131.06 (1.46)a	52.03 (0.99)b	44.38 (0.07)c	118.64 (1.05)a	67.49 (0.72)c	92.59 (1.43)b
**GalA**	6.75 (0.27)a	3.17 (0.02)c	5.01 (0.06)b	6.36 (0.10)a	6.94 (0.06)a	5.45 (0.29)b
**GlcA**	N.d	N.d	N.d	N.d	N.d	N.d
**Total sugars**	478.79 (8.06)	211.55 (3.66)	170.19 (0.22)	349.35 (3.12)	257.76 (2.99)	328.25 (6.17)
High-firmness varieties						
Variety	Draper					
Sugars	Pre-veraison	Veraison	Harvest			
**Fuc**	2.87 (0.05)a	1.58 (0.06)b	1.70 (0.10)b			
**Rha**	3.50 (0.03)a	3.74 (0.11)a	2.18 (0.01)b			
**Ara**	57.52 (0.59)a	45.14 (0.50)b	24.43 (0.93)c			
**Gal**	67.14 (0.81)a	54.16 (0.72)b	48.06 (1.63)c			
**Glc**	147.64 (2.18)a	91.14 (1.23)b	79.09 (2.72)c			
**Man**	30.49 (0.23)a	24.98 (0.59)b	17.46 (0.60)c			
**Xyl**	133.65 (1.01)a	89.24 (0.84)b	70.50 (2.19)c			
**GalA**	5.16 (0.30)b	9.40 (0.34)a	4.81 (0.08)b			
**GlcA**	N.d	N.d	N.d			
**Total sugars**	447.96 (5.11)	319.56 (3.96)	248.22 (8.25)			

Sugar contents were determined for hemicellulose fractions. Values represent the means and SE (in parentheses) of three technical repeats (n = 3). Letters denote differences between different monosaccharides for each variety with statistical analyses performed using the Tukey test. Data were compared between each variety with p < 0.0001. N.d, corresponds to not detected sugar.

To investigate whether changes in sugar content could affect the structure of xylans, arabinoxylans, or xyloglucans, the Ara/Xyl ratio (related to arabinoxylans) and Glc/Xyl ratio (related to xyloglucans) were calculated at harvest (**Figure 10D**). The Ara/Xyl ratio showed a trend of decreasing from low-firmness varieties to high-firmness varieties.The Glc/Xyl ratio remained consistent for low-firmness varieties but decreased for medium-firmness varieties, although Draper exhibited a ratio similar to Bluecrop and Legacy. Dot blot assays were performed on purified HC fractions from all varieties at three ripening stages using different antibodies that recognize xylans and arabinoxylans (**Supplementary Figures 2**, **3**). Detection of xylan with LM10, LM11, and CCRC-M139 antibodies (McCartney et al., 2005; Ruprecht et al., 2017; Pattathil et al., 2010) yielded similar results for all three antibodies. A decrease in labeling intensity was observed during development in all varieties except Duke, which showed only subtle variations in labeling intensity from pre-veraison to harvest. Anti-xylan antibodies did not reveal differences between varieties and their firmness throughout ripening (**Supplementary Figures 2**, **3**). Detection of arabinoxylan with AX1 antibody (Guillon et al., 2004) revealed a significant decrease in the AX1 epitope during softening in the Legacy, Brigitta, Duke, and Draper varieties, with a less pronounced change in Bluecrop, suggesting that arabinoxylan structure might be involved in fruit firmness.

**Figure 11:**
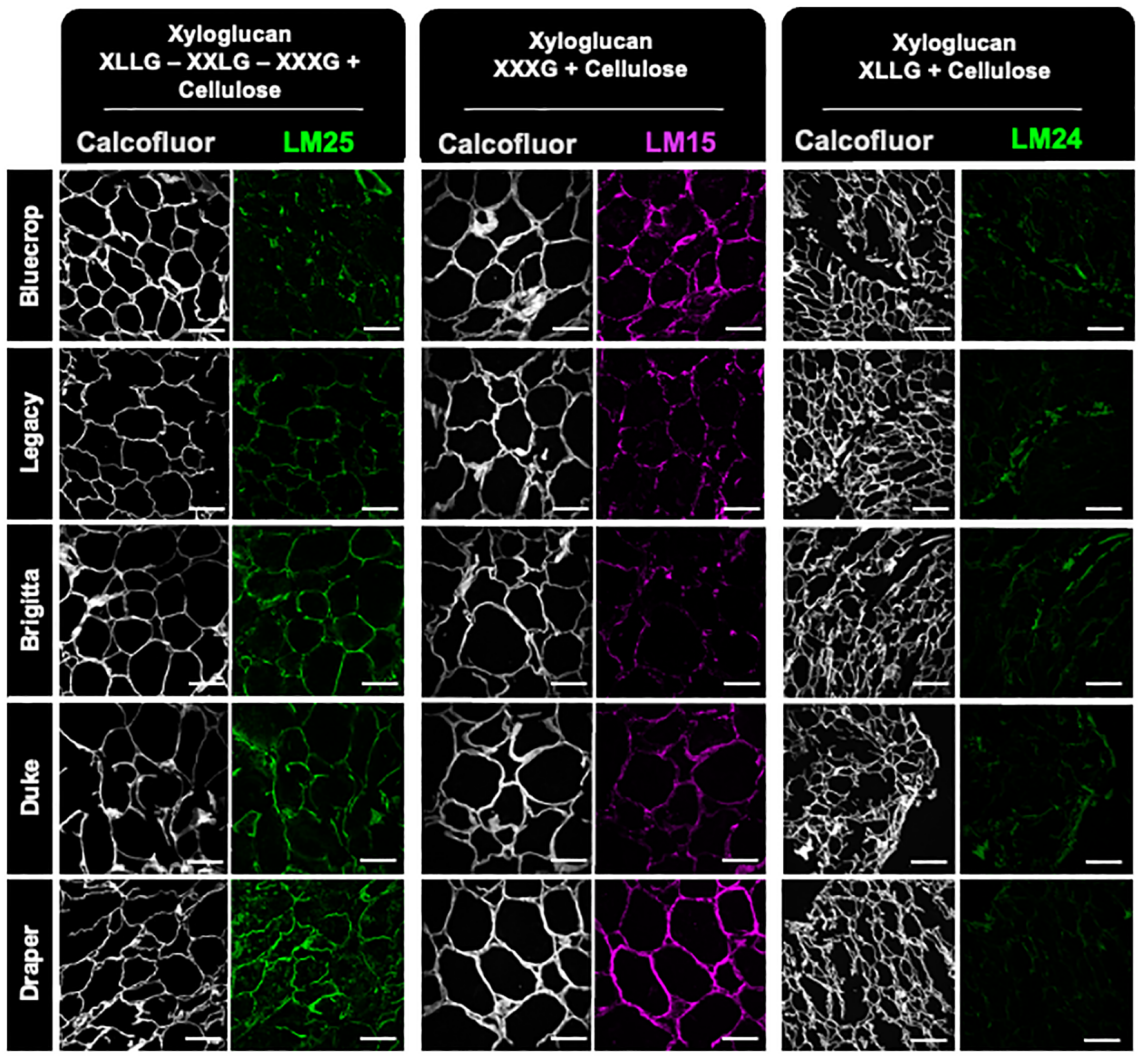
Immunolabeling analysis of xyloglucan epitopes in fruit sections at the harvested stage reveals an increased label for the firmer variety. Sections from mature blueberry fruit were imaged using confocal microscopy to reconstruct optical sections. Immunolabeling was performed using LM25, LM15, and LM24 antibodies to specifically label xyloglucan. These labels were visualized in green or magenta fluorescence. Simultaneously, calcofluor white, which detects beta-1,4-glucans, was used and appeared as gray fluorescence. The scale bar represents 100 μm.

The analysis of xyloglucan epitopes was performed on fruit sections immunolabeled with LM15 (XXXG), LM24 (XLLG), and LM25 (XLLG, XXLG, and XXXG) (Moller et al., 2008; Pedersen et al., 2012) (**Figure 11**). The LM24 antibody, which recognizes the xyloglucan epitope XLLG, shows minimal labeling, particularly diminishing in the firmer Duke and Draper varieties. In contrast, LM15, which targets the XXXG backbone, showed strong labeling in Bluecrop and Draper. The LM25 antibody displayed a labeling profile closely correlated with fruit firmness, with signal intensity increasing progressively from the low-firmness to the medium-firmness variety, and ultimately to the firmest variety. These differences in epitope recognition suggest that the xyloglucans in the various varieties might have distinct substitution patterns.

## 4 Discussion

The present study examined fruit firmness in five commercial blueberry cultivars: Bluecrop, Legacy, Brigitta, Duke, and Draper. These cultivars exhibited a range of firmness levels, from the softest, Legacy (198,6N), to the firmest, Draper (341.3 N). This variation in firmness among blueberry varieties provides a valuable opportunity to investigate the role of the PCW in fruit firmness. The PCW is a complex network of polysaccharides, including cellulose, HCs, pectins, and AGPs, which provide structural support to the cell. Understanding the composition, structure, and interactions of these PCW components is essential for enhancing fruit quality, meeting consumer preferences, and extending fruit shelf life.

A notable finding is the decrease in total sugar content in the AIR during fruit ripening in the softer and medium-firmness varieties (Bluecrop, Legacy, Brigitta, and Duke). In contrast, the firmest variety, Draper, exhibited only a slight and statistically insignificant decrease in total sugar content. These findings highlight the importance of maintaining cell wall integrity to preserve a stable network during fruit ripening, thereby ensuring fruit firmness. However, total sugar content in the AIR does not fully elucidate changes in cell wall structure during ripening. Detailed analysis of polysaccharide structure is needed to comprehensively understand the role of the cell wall in fruit ripening.

Monosaccharide composition analysis of both the AIR and separately pectin- and HC-enriched fractions revealed distinct results. GalA, a predominant sugar in blueberries, showed minimal changes in AIR content, except for an increase in Legacy and a decrease in Brigitta during ripening. Immunohistochemical analysis using JIM5 and 2F4 antibodies on fruit sections at the harvest stage revealed alterations in HG distribution and/or the methylesterification pattern, not necessarily linked to egg-box structure formation within the cell wall. However, changes in HG patterns consistently correlate with cell wall stiffness. This is evident from the differential labeling intensity of JIM5 and 2F4 between Bluecrop and Draper, with Draper showing high detection of demethylesterified pectin and egg-box structures, while Bluecrop exhibited intense labeling for low-methylated HG but minimal 2F4 signal. Therefore, using JIM5 alone does not provide information on HG methylation patterns, as it targets specific epitopes. HG metabolism is complex, and random demethylation, depending on the methylation pattern, can render HG a suitable substrate for pectin-degrading enzymes such as PGs, thereby weakening the cell wall (Wormit and Usadel, 2018).

No changes in GalA content were observed in the enriched pectin fraction between firmer and softer fruit. However, the monosaccharide composition does not accurately reflect the pectin structures. Vicente et al. (2007) reported that ripening-associated softening in blueberries involves solubilization of pectin at early and intermediate stages of ripening, without significant pectin depolymerization at late ripening stages. The positive effects of cross-linking on firmness retention may arise from reduced pectin solubilization due to the formation of calcium bridges or an indirect effect on HC disassembly, rather than the prevention of pectin depolymerization. These findings are consistent with previous knowledge, suggesting egg-box structures through calcium-ion-mediated cross-linking of demethylesterified pectins is a crucial mechanism for enhancing cell wall stiffness (Ngouémazong et al., 2012).

Interestingly, the analysis of GalA content in the pectin fraction does not correlate with the results from the AIR analysis. This discrepancy may arise from the differing complexities of matrices such as AIR and pectins; AIR is notably more complex and may interfere somewhat with the acid hydrolysis process. A concept closely tied to GalA is methanol content, as GalA can exist in methylated or unmethylated forms, significantly impacting cell wall stiffness. Analysis of methanol quantification in the pectin fraction confirmed a decrease in methanol content during fruit ripening, providing additional evidence supporting the link between reduced pectin methylesterification, increased egg-box structures, and fruit firmness.

While GalA and Xyl are the primary sugar components of blueberry cell walls, with their roles in firmness confirmed (Olmedo et al., 2021), other polysaccharides present in lower abundance within the cell wall may also play relevant roles. Blueberry cell walls contain relatively low amounts of rhamnose, a sugar constituting the RG-I backbone, suggesting that this domain is not prevalent in blueberry cell walls. Despite its low abundance, modifications to RG-I have been identified as a significant contributor to cell wall density (Peña and Carpita, 2004; Arsovski et al., 2009; Morales-Quintana et al., 2022). Changes in monosaccharide composition were detected in both AIR and pectin fractions, indicating depolymerization of RG-I side chains during ripening. However, despite consistency between AIR and pectin analyses, concerns arose due to sugars originating from HCs in the AIR and the co-extraction of pectin and AGPs. This suggests a potential linkage between them, similar to the covalent bonds detected between AGPs and RG-I in Arabidopsis cells (Tan et al., 2023a, 2024). The association between AGPs and RG-I in relation to fruit firmness remains uncertain, prompting further investigation. To address these concerns, RG-I was isolated using size exclusion chromatography and subjected to monosaccharide composition analysis and dot blot assays, alongside fruit sections (resin-imbibed) for immunolabeling. Monosaccharide analysis of isolated RG-I revealed a trend in the Ara/Rha ratio, decreasing in medium-firmness varieties and increasing in the firmest and softest varieties. This finding, not fully reflected in pectin structure, was better understood using antibodies, which provided insights into the RG-I structure. The detection of the RG-I backbone (INRA-RU1) showed increasing label intensity correlating with firmness, suggesting the presence of an unbranched domain associated with fruit firmness. This observation was supported by more pronounced labeling in the galactan side chains (LM5) in the softest varieties, significant due to their role in water-binding capacity (WBC). Studies in potatoes have shown that modifications to RG-I β-(1→4)-D-galactan side chains can reduce WBC (Klaassen and Trindade, 2020), highlighting the importance of subtle changes in the RG-I structure on cell wall properties. In Arabidopsis mucilage mutant lines affected in the lateral chains of RG-I, defects in cell wall breakage and polysaccharide hydrophobicity have been observed, confirming that even subtle changes in RG-I structure can significantly impact cell wall stiffening (Macquet et al., 2007b; Saez-Aguayo et al., 2014). These subtle changes in RG-I lateral chain structure effect could possibly stem from changes in polysaccharide architecture (spacing) and interactions within the matrix.

Analyzing sugar ratios in pectin-enriched fractions, we observed that Gal/Rha and Ara/Rha ratios were lowest at harvest in the Draper variety, indicating a higher proportion of Rha compared to Gal and Ara. Trandel-Hayse et al. (2023) reported that an increased ratio of Ara/Rha suggests a higher presence of hairy side branches, which could lead to increased pectin solubilization and softening. Combined with immunolabeling results showing intense labeling of the RG-I backbone and minimal galactan side branches in Draper, these results suggest that firmer fruits like Draper may have RG-I with fewer or shorter side branches. Since arabinose and galactose form the neutral sugar side chains of RG-I, which can be covalently linked to HC and cellulose, their removal and/or rearrangement greatly influences cell wall strength and porosity (Brummell, 2006; Guillon et al., 2017; Ng et al., 2015; Chea et al., 2019).

The AGP analysis, conducted by dot blot using isolated RG-I and immunolabeling in fruit slices, showed a lower label intensity in Draper. These results suggest the involvement of RG-I and AGPs in fruit firmness. When comparing the variations in Ara and Gal content and their ratios relative to Rha in the pectin-enriched fraction and isolated RG-I, the results differ. For the isolated RG-I, Draper and the softest varieties show similar results, whereas for the pectin-enriched fraction, Draper shows the lowest levels among all varieties. This suggests that other structures chemically co-extracted with pectins may contribute to the measured amounts of Ara and Gal. It has been reported that arabinogalactan type-II chains of AGPs can be linked to RG-I (Tan et al., 2023a; 2024). The role of AGPs as cross-linkers emphasizes their potential in forming a continuous network between wall polysaccharides and structural proteins in the cell wall (Tan et al., 2013; 2024). These results support our hypothesis that fruit firmness is not solely due to HG demethylesterification, egg-box structures, and HC modifications but also involves RG-I and AGPs in a variety-dependent manner. Shorter and/or less-branched RG-I may affect its linkage to AGPs, influencing cell wall densification. Further validation with additional separation techniques and antibodies like LM2 and Yariv's reagent will be advantageous for confirming the association between AGPs and RG-I.

Hemicelluloses interact with cellulose fibers, playing a significant role in maintaining cell wall integrity. A decrease in HC content is associated with softening, though different varieties and species exhibit distinct behaviors. Analyses revealed that 4 out of 5 varieties showed a significant decrease in HC content between pre-veraison and veraison, with marked reduction in Bluecrop and Brigitta during this period, and a milder transition from veraison to harvest. Vicente et al. (2007) reported increased solubilization of the pectic matrix at early and intermediate ripening stages, with no significant changes in late ripening. For instance, in tomatoes, HC involvement in fruit softening was not evident from compositional analysis, but polymer size analysis revealed a decrease in size associated with softening (Huber, 1983). In contrast, HC levels decreased as ripening progressed, with clear depolymerization. Legacy and Duke reached harvest with HC values close to their firmer condition. Chea et al. (2019) previously described a similar situation specifically for Bluecrop. Draper exhibited a proportional decrease in HC content during softening, with no pronounced differences between stages, highlighting the significance of HC depolymerization in blueberry fruit softening.

Xylose is highly abundant in blueberries, and its variations during ripening or among varieties are noteworthy. An intriguing pattern emerged in its profile, with Xyl content decreasing during ripening in most varieties, except for Draper, the firmest one. Notably, Draper consistently maintained its Xyl content, underscoring the significant role of HC in determining fruit firmness. Draper is the only variety that exhibits almost no variation in the amount of Xyl during ripening, alongside a decrease in the methylated HG. Considering the presence of egg-box structures and the behavior of xylose during ripening in Draper, it is plausible that this polymer might also contribute to cell wall stiffening. Hemicelluloses consist of several domains containing Xyl, such as xylan, arabinoxylan and xyloglucan. The predominance of Xyl and Glc in the HC-enriched fraction suggests xyloglucan as a primary HC component. Xyloglucan consists of a backbone of Glc residues, most substituted with Xyl side chains. Xylose residues in xyloglucan are often capped with a Gal, sometimes followed by a Fuc residue. It is important to note that the specific structure of xyloglucan can vary across different plant families. The Glc/Xyl ratio in the HC fraction did not align with the expected 4:3 (1.33) ratio for xyloglucan but showed Xyl exceeding Glc, suggesting a mixture of xyloglucan and other polymers like xylan and arabinoxylans in the HC fraction (Vicente et al., 2007).

When examining the Glc/Xyl ratio across different varieties, no consistent pattern emerges based on firmness characteristics. For instance, both softer varieties showed a decrease in the ratio from pre-veraison to harvest, while medium-firmness berries exhibited the opposite trend. Duke showed a decrease in the ratio, while Brigitta showed an increase. Notably, Draper maintained a constant ratio, reinforcing the concept that preserving the HC network significantly contributes to fruit firmness. The involvement of xyloglucan in blueberry firmness has been reported by Trandel-Hayse et al. (2023). They found that the higher firmness in the pulp of “Indigocrisp” and “Emerald” varieties was associated with a higher content of xyloglucan. A decrease in the Glc/Xyl ratio implies an excess of Xyl compared to the amount necessary to form the basic backbone of xyloglucan (XXXG). This suggests the presence of other polymers composed of Xyl, such as xylan. Blueberries have been reported to contain short sclereids (Fava et al., 2006), also known as stone cells, which are non-living cells with thick secondary cell walls rich in xylan. This could explain the observed high Xyl content.

Another aspect that could provide additional insights into the involvement of xyloglucan in fruit firmness lies in the analogous behavior observed in the immunolabeling performed by JIM5 (low-methylated HG) and LM15 (XXXG in xyloglucan), as well as by 2F4 (egg-box) and LM25 (XXXG, XLLG, and XXLG). Regarding JIM5 and LM15, both experiments show more intense signals in Bluecrop (the softest) and Draper. For 2F4 and LM25, the most intense signal was detected in Draper. Taken together, these results suggest that xyloglucan, in combination with other polymers, may play a significant role in fruit firmness and needs to be studied extensively.

## 5 Conclusion

In conclusion, the comparative analysis of cell wall components across five blueberry varieties with varying firmness profiles has reinforced the pivotal role of the egg-box structure in maintaining cell wall rigidity. Although the exact contribution of HC remains somewhat elusive, it appears that xyloglucan integrity also plays an important role. Moreover, our study has unveiled the participation of the RG-I structure associated with AGPs in fruit firmness. These results enhance our understanding of cell wall dynamics in firm blueberries, characterized by significant modifications in pectin and HC components. In essence, firmer fruit exhibit the following characteristics: (1) limited HC depolymerization during fruit ripening of xyloglucans; (2) a denser pectin matrix with extensively demethylesterified HG, resulting in increased egg-box structures; and (3) the presence of RG-I with shorter or fewer branches leading to impaired interaction with AGPs. It is worth noting that further comprehensive analyses are warranted to fully unravel the specific roles of HC and cellulose in influencing fruit firmness.

## Data Availability

The original contributions presented in the study are included in the article/**Supplementary Material**. Further inquiries can be directed to the corresponding author.
