# Supplementary material for: Unraveling cell wall polysaccharides during blueberry ripening: insights into the roles of rhamnogalacturonan-I and arabinogalactan proteins in fruit firmness
Source: Front Plant Sci. 2024 Sep 30;15:1422917. doi: 10.3389/fpls.2024.1422917 (PMC11472824; doi:10.3389/fpls.2024.1422917)
Supplement: Supplementary file 1 [file Presentation1.pdf]

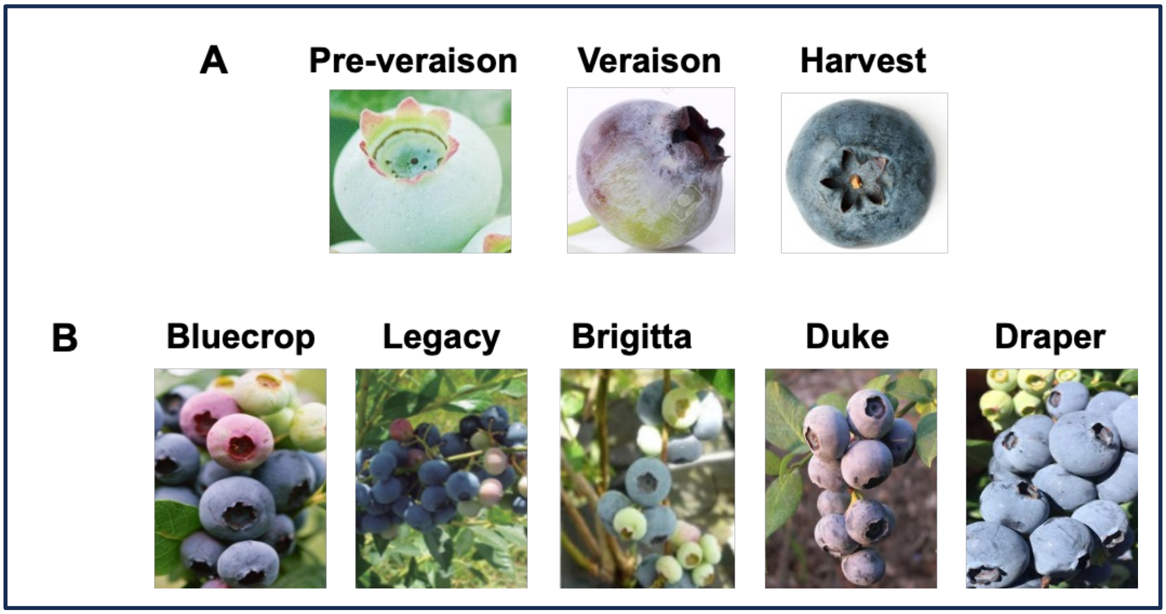

Supplementary figure 1: Representation of the three developmental stages and various blueberry varieties studied. A. Illustration of the three developmental stages examined in this study. **B.** Photographs of the five blueberry varieties analyzed in this study.

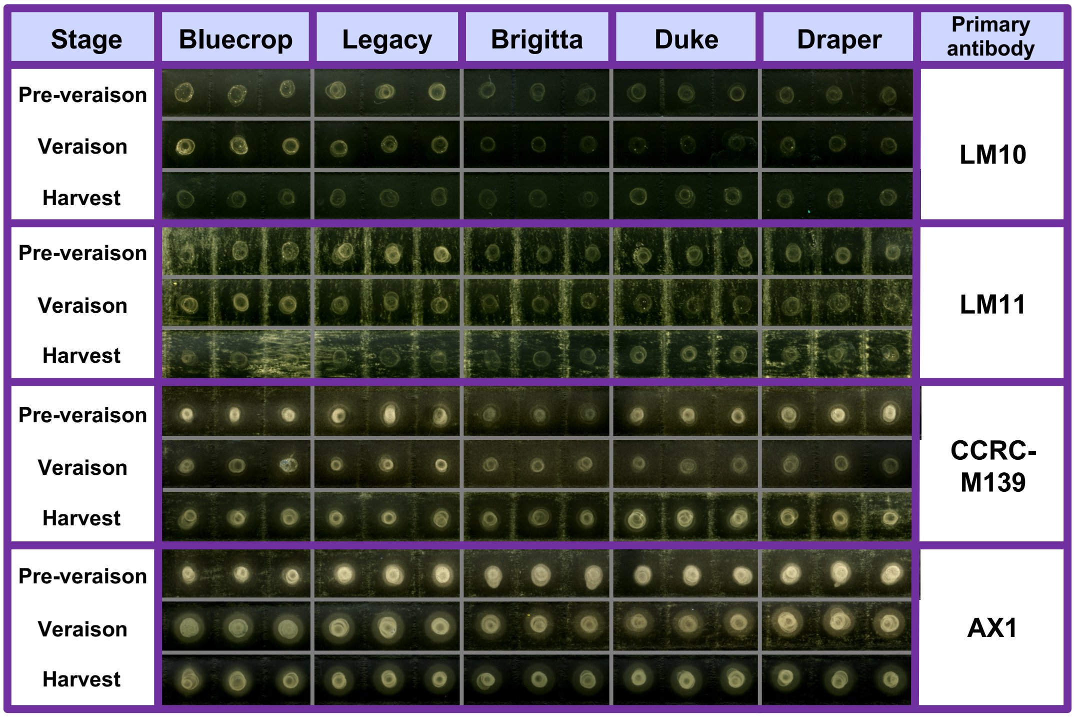

Supplementary figure 2: Analysis of xylan and arabinoxylan epitopes in purified hemicellulose fractions.

Dot blot analysis was conducted using purified hemicellulose fractions from all samples during fruit ripening. Various antibodies were applied to detect xylan (CCRC-M139, LM10, LM11), and arabinoxylan (AX1). The analysis of xylan and arabinoxylan did not allow a discernible pattern related to their labeling concerning fruit firmness.

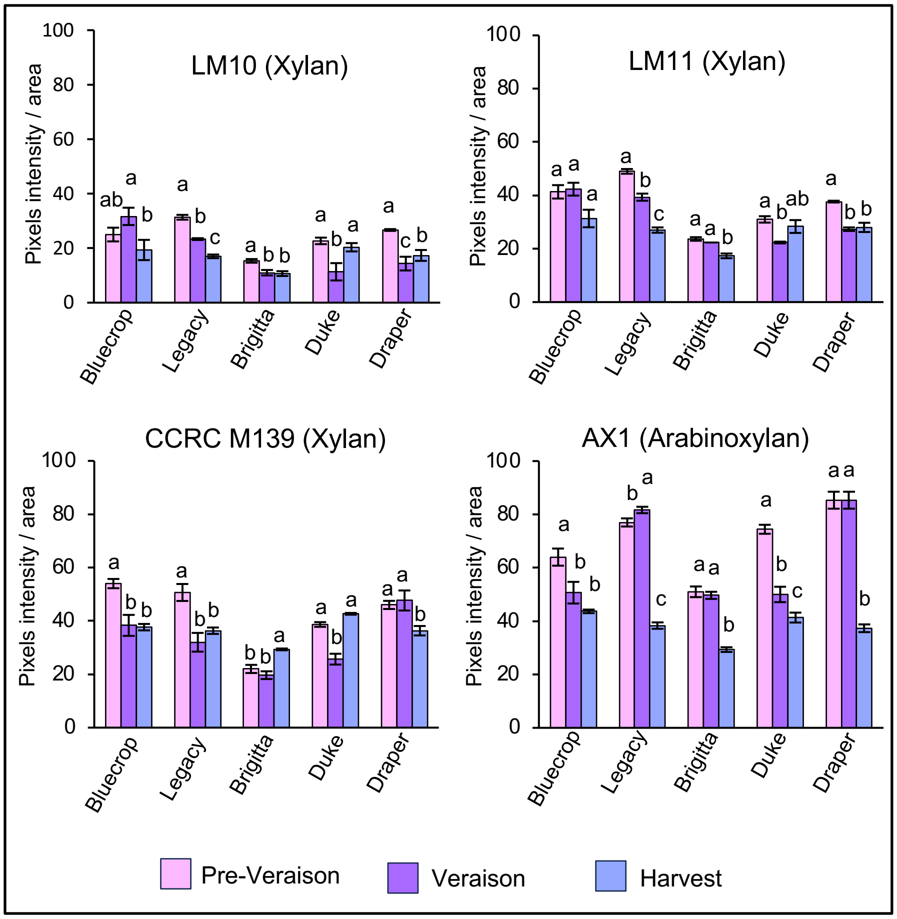

Supplementary figure 3: Quantification of pixels in the hemicellulose dot blot analysis.

The standard error (SE) was calculated from three replicates. Statistical analysis included One-way ANOVA and pairwise Student´s t-test comparison. Letters indicate significant differences between stages for each variety with $p < 0.05$.
